# Recent Advances in Strategies to Enhance Photodynamic and Photothermal Therapy Performance of Single‐Component Organic Phototherapeutic Agents

**DOI:** 10.1002/advs.202409157

**Published:** 2025-01-10

**Authors:** Laiping Fang, Zengzhen Chen, Jianan Dai, Yujin Pan, Yike Tu, Qi Meng, Yanzhao Diao, Shuaibo Yang, Wei Guo, Liming Li, Jinwu Liu, Hua Wen, Kelei Hua, Lifeng Hang, Jin Fang, Xianwei Meng, Ping'an Ma, Guihua Jiang

**Affiliations:** ^1^ Guangdong Second Provincial General Hospital School of Medicine Jinan University Xingangzhong Road 466 Guangzhou 518037 P. R. China; ^2^ State Key Laboratory of Cryogenic Science and Technology Technical Institute of Physics and Chemistry Chinese Academy of Sciences Zhongguancun East Road 29 Beijing 100190 P. R. China; ^3^ College of Information Technology Jilin Normal University Haifeng Street 1301 Siping 136000 P. R. China; ^4^ Department of Hepatobiliary and Pancreatic Surgery Henan Provincial People's Hospital Weiwu Road 7 Zhengzhou 450003 P. R. China; ^5^ The Department of Medical Imaging The Affiliated Guangdong Second Provincial General Hospital of Jinan University Xingangzhong Road 466 Guangzhou 518037 P. R. China; ^6^ State Key Laboratory of Rare Earth Resource Utilization Changchun Institute of Applied Chemistry Chinese Academy of Sciences Renmin Street 5625 Changchun 130012 P. R. China

**Keywords:** single‐component organic phototherapeutic agents, photodynamic therapy, photothermal therapy

## Abstract

Photodynamic therapy (PDT) and photothermal therapy (PTT) have emerged as promising treatment options, showcasing immense potential in addressing both oncologic and nononcologic diseases. Single‐component organic phototherapeutic agents (SCOPAs) offer advantages compared to inorganic or multicomponent nanomedicine, including better biosafety, lower toxicity, simpler synthesis, and enhanced reproducibility. Nonetheless, how to further improve the therapeutic effectiveness of SCOPAs remains a challenging research area. This review delves deeply into strategies to improve the performance of PDT or PTT by optimizing the structural design of SCOPAs. These strategies encompass augmenting reactive oxygen species (ROS) generation, mitigating oxygen dependence, elevating light absorption capacity, broadening the absorption region, and enhancing the photothermal conversion efficiency (PCE). Additionally, this review also underscores the ideal strategies for developing SCOPAs with balanced PDT and PTT. Furthermore, the potential synergies are highlighted between PDT and PTT with other treatment modalities such as ferroptosis, gas therapy, chemotherapy, and immunotherapy. By providing a comprehensive analysis of these strategies, this review aspires to serve as a valuable resource for clinicians and researchers, facilitating the wider application and advancement of SCOPAs‐mediated PDT and PTT.

## Graphical abstract

Single‐component organic phototherapeutic agents (SCOPAs) for photodynamic therapy and photothermal therapy hold promise in treating diseases. This review delves into strategies for enhancing SCOPAs performance, such as boosting ROS generation, improving the photothermal conversion efficiency, and summarizes potential synergies with other therapeutic modalities, aiming to promote the transition of SCOPAs toward broad clinical applications.

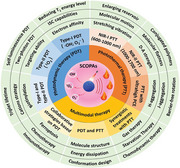

## 1 Introduction

Cancer is a disease caused by abnormal cell proliferation, leading to the formation of tumors through uncontrolled cell growth.^[^ ^1^ ^]^ In 2020, there were 19.29 million new cancer cases and 9.96 million cancer‐related deaths worldwide, as reported by the International Agency for Research on Cancer.^[^ ^2^ ^]^ Over the years, advancements in cancer diagnosis and treatment have been continually evolving. From the earliest cases documented in ancient Egypt and Greece, to the first excision surgery in the 18th century, and the introduction of radiotherapy in the late 19th century, human efforts to understand and combat cancer have been ongoing.^[^ ^3^ ^]^

**Scheme 1:**
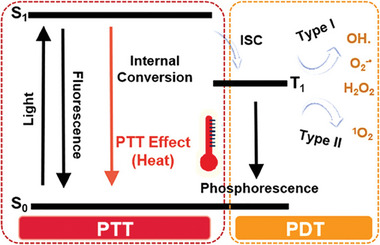
Schematic of a Jablonski diagram to present the mechanism of PDT and PTT. Reproduced with permission. Reproduced with permission.^[^ ^14^ ^]^ Copyright 2022, American Chemical Society.

Chemotherapy, surgery, and radiotherapy are the three conventional means of treating cancer.^[^ ^4^ ^]^ However, chemotherapy is limited in its clinical efficacy due to factors such as poor water solubility, low selectivity for cancer cells, high drug resistance, and low maximum tolerated dose of drugs, often leading to severe adverse reactions.^[^ ^5^, ^6^ ^]^ Surgical resection is a high‐risk invasive treatment. As a local therapy, it is only effective for nonmetastatic tumors, facing the risk of incomplete resection and tumor recurrence.^[^ ^7^ ^]^ Radiotherapy, while using radiation to target cancer cells, also poses a threat to normal tissues and cells.^[^ ^8^ ^]^ In recent years, phototherapy, including PDT and PTT, has attracted widespread attention from researchers due to its noninvasiveness, spatiotemporal controllability, and low toxic side effects.^[^ ^9^, ^10^, ^11^ ^]^ Phototherapy mainly relies on a series of photophysical transformation processes after the phototherapeutic agent absorbs photons and transitions from the ground to the excited state. The excited phototherapeutic agent emits photons through radiative transition for fluorescence or phosphorescence imaging, generates heat for PTT after returning to the ground state through nonradiative transitions, and produces ROS for PDT by transitioning to the triplet state. When light energy is converted into chemical or thermal energy, it can trigger a series of reactions that induce tumor cell death (**Scheme** **1**).^[^ ^12^, ^13^, ^14^ ^]^

PDT induces necrosis and apoptosis by generating highly toxic ROS through energy or electron transfer mechanisms.^[^ ^15^, ^16^, ^17^ ^]^ Currently, hundreds of photosensitizers (PSs) are undergoing clinical trials or are already in clinical use, including photofrin, foscan, talaporfin, and radachlorin.^[^ ^18^, ^19^, ^20^ ^]^ For over 40 years, PDT has been utilized in the treatment of various cancers, including bladder cancer, esophageal cancer, lung cancer, and certain nonmelanoma skin cancers.^[^ ^21^ ^]^ On the other hand, PTT transforms light into heat through photothermal reagents (PTAs), aiming to raise the temperature at the tumor site to achieve therapeutic objectives.^[^ ^22^, ^23^, ^24^ ^]^ Preclinical studies and initial trials of PTT have shown promising therapeutic outcomes for superficial tumors like skin, head, and neck cancers.^[^ ^25^, ^26^ ^]^ Approved PTAs for clinical tumor treatment include gold nanoshells and indocyanine green (ICG).^[^ ^27^ ^]^ It's worth noting that most existing PTAs are inorganic metal nanomaterials, including black phosphorus, titanium dioxide, CuS, transition metals, and 2D organic metal framework materials, all of which exhibit dose‐dependent toxic side effects.^[^ ^28^, ^29^, ^30^, ^31^, ^32^ ^]^ Repeated administration poses the risk of cumulative toxicity, limiting their widespread application in the biological field. SCOPAs, including cyanine dyes, are widely utilized in biological applications due to their diverse structures, adjustable performance, and good safety profile. These SCOPAs have demonstrated significant phototherapeutic effects in processes of PDT and PTT. Despite their effectiveness, challenges such as stability issues, limited production of type‐I ROS, and the need to enhance PCE persist.^[^ ^33^ ^]^ Moreover, the combination of phototherapy with other modalities like ferroptosis, cuproptosis, immunotherapy, and chemotherapy is emerging as a promising approach in tumor treatment. This multimodal therapy strategy aims to address the limitations of single‐modality treatments and achieve a synergistic antitumor effect greater than the individual components.^[^ ^34^, ^35^, ^36^, ^37^, ^38^ ^]^

**Scheme 2:**
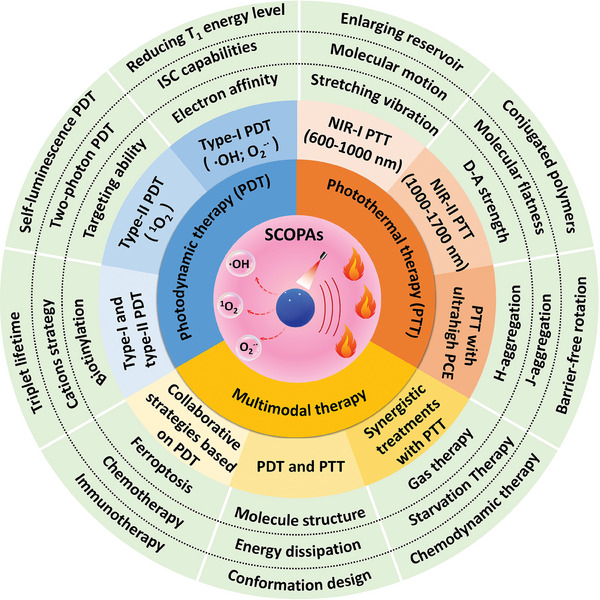
Schematic diagram of kinds of adaptive improvement strategies for SCOPAs‐based PDT and PTT and the synergistic therapies with other methods.

So far, the use of SCOPAs for near‐infrared fluorescence imaging (NIR‐FLI)‐guided multimodal synergistic phototherapy is a current cutting‐edge research area. While progress has been made in designing, synthesizing, and exploring the structure‐activity relationships of SCOPAs, as well as their biomedical applications, there is a need for a comprehensive summary of strategies to enhance the efficiency of PDT and PTT of SCOPAs. This paper will focus on strategies to improve type‐II PDT, promote type‐I PDT, and combine type‐I/II PDT, as well as explore multimodal collaborative treatments based on PDT. Additionally, it will discuss strategies to enhance NIR‐I photothermal conversion, develop NIR‐II PTAs, and develop PTAs with ultra‐high PCE in the PTT section, along with applied research on multimodal collaborative treatments based on PTT. Furthermore, methods to design SCOPAs capable of simultaneous PDT and PTT performance will be presented in the PDT synergistic PTT section. Finally, the paper will address the prospects and challenges of applying SCOPAs‐mediated PDT and PTT in clinical settings (**Scheme** **2**).

## 2 Photodynamic Therapy

As a novel cancer treatment modality, PDT combines several advantages such as minimal invasiveness, high efficiency, and controllability, and has already made its mark in clinical healthcare.^[^ ^39^ ^]^ Depending on the type of ROS generated by photodynamic reaction (PDR), PDT can be categorized into type‐I PDT and type‐II PDT. The PDR of type‐I PDT involves the electron/hydrogen transfer between triplet state (T_1_)‐PSs and oxygen, producing cytotoxic ROS such as superoxide anion (O_2_^•−^), hydroxyl radical (•OH), and hydrogen peroxide (H_2_O_2_). On the other hand, the PDR of type‐II PDT involves energy transfer from the T_1_ state‐PSs to triplet oxygen, resulting in the generation of singlet oxygen (^1^O_2_), which promotes cell necrosis and apoptosis.

Type‐II PDT is the most extensively studied form of PDT and primarily relies on the highly reactive and oxidizing species ^1^O_2_ for treating diseased tissue. This species has strong electrophilicity and can efficiently oxidize various components such as unsaturated fatty acids, nucleic acids, proteins, and mitochondrial membranes, ultimately leading to tumor cell death.^[^ ^40^, ^41^ ^]^ However, the effectiveness of type‐II PDT is limited by the availability of oxygen, as low oxygen concentrations hinder the production of significant amounts of ^1^O_2_. This oxygen dependency presents challenges, particularly in the tumor microenvironment (TME), where increased oxygen consumption during treatment can worsen tumor hypoxia, leading to enhanced tumor growth, metastasis, and invasion, thus affecting therapeutic outcomes.^[^ ^42^, ^43^ ^]^ On the other hand, type‐I PDT has a lower reliance on oxygen, as it can generate ROS like O_2_^•−^ and •OH through disproportionation and Harber–Weiss/Fenton reactions even under severe hypoxia conditions (2% O_2_), providing a significant advantage in treating solid tumors.^[^ ^44^ ^]^ Although type‐I PDT reduces the dependence on oxygen, it still needs oxygen to generate ROS. Therefore, Peng et al. introduced the idea of type‐III PDT, where PSs target proteins, nucleic acids, and other biomacromolecules in cells. By directly damaging these targets upon binding, type‐III PDT effectively addresses the issue of low oxygen levels in traditional PDT, providing a potential solution for enhancing phototherapy in deep‐seated tumors.^[^ ^45^ ^]^

The vast potential of PDT has driven the rapid development of SCOPAs. Traditional SCOPAs like porphyrins, phthalocyanines, and phenothiazines have been widely used in clinical treatments.^[^ ^46^, ^47^, ^48^, ^49^ ^]^ Their large planar structures may lead to aggregation‐induced quenching (ACQ), limiting their ROS generation capability.^[^ ^50^, ^51^ ^]^ However, this process is often reversible upon cell uptake and nanoparticle dissociation. In fact, it can be even an asset since quenching can limit PDT off‐target toxicity and be leveraged for site‐ and time‐dependent activation. Moreover, since quenched structures often have higher PCE this lends itself for dual PTT‐PDT agents.^[^ ^52^, ^53^, ^54^, ^55^, ^56^ ^]^ Not only that, the recent advancements in nanotechnology have also promoted the development of nanobased SCOPAs, such as aggregation‐induced emission (AIE)‐based NPs, which exhibit enhanced photostability and ROS generation efficiency through molecular design.^[^ ^57^, ^58^, ^59^, ^60^, ^61^, ^62^, ^63^, ^64^ ^]^

### 2.1 Strategies to Improve Type‐II PDT

Type‐II PDT primarily relies on the excited electrons undergoing intersystem crossing (ISC) to transfer their energy to surrounding oxygen molecules, generating ^1^O_2_. The interaction of ^1^O_2_ with cancer cells leads to the destruction of cell membranes or vital proteins, ultimately killing the cells. Generally, the speed of type‐II PDT is typically faster than that of type‐I PDT. When the energy levels meet the requirements, PDT tends to favor type‐II over type‐I. Consequently, most of the commercialized and in‐development PSs are type‐II PSs. Given that the hypoxic TME, how to improve the therapeutic effects of type‐II PDT is more significant.

#### 2.1.1 Improving the Production Efficiency of ^1^O_2_

Cyanine‐based SCOPAs have found widespread applications in the biomedical field, but how to improve the ^1^O_2_ production efficiency of cyanine has not been clearly studied.^[^ ^65^ ^]^ In light of this, Li and his colleagues systematically investigated anionic cyanines based on the strongly electron‐deficient tricyanofuran (TCF) terminal group, discovering that the counterion plays a crucial role in molecular assembly and optical properties. They successfully synthesized the first TCF‐based anionic cyanine photosensitizer, called C3T‐Pc, utilizing the mitochondrial targeting agent dodecyl(triphenyl)phosphine cation (Pc) as the counterion. Interestingly, C3T‐Pc could form supermolecular J‐aggregates in aqueous solutions that exhibited pronounced red‐shifted emission, negatively charged surfaces, and high photostability. Moreover, its ^1^O_2_ generation efficiency was five times higher than that of nonaggregated forms. Theoretical calculations revealed that this enhancement was attributed to the J‐aggregation‐induced reduction of singlet‐triplet energy gap (ΔE_S‐T_), which facilitated ISC.^[^ ^66^ ^]^

**Figure 1:**
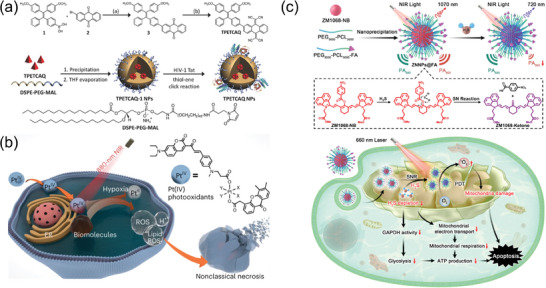
a) Synthesis of TPETCAQ and preparation of TPETCAQ NPs. Reproduced with permission.^[^ ^68^ ^]^ Copyright 2017, Wiley‐VCH. b) Pt^IV^ can oxidize biomolecules under NIR light irradiation by generating ROS without the need for oxygen. Reproduced with permission.^[^ ^70^ ^]^ Copyright 2023, Springer Nature. c) The fabrication of ZNNPs@FA and consumption of endogenous H_2_S in colorectal cancer inhibits ATP synthesis and activates the photodynamic effect of ZNNPs@FA. Reproduced with permission.^[^ ^82^ ^]^ Copyright 2023, Springer Nature.

Additionally, the preparation of AIE‐type SCOPAs is also an effective strategy to overcome the low photosensitivity efficiency of traditional SCOPAs.^[^ ^67^ ^]^ The Liu research group designed the first photosensitizer with aggregation‐induced near‐infrared fluorescence emission, named TPETCAQ (**Figure** **1**a).^[^ ^68^ ^]^ Further formulated into NPs, these NPs exhibited a broad absorption range and the fluorescence emission wavelength exceeding 820 nm. Under white light irradiation, TPETCAQ NPs could generate more ^1^O_2_ than the commercial Ce6 and demonstrate highly efficient photodynamic killing effects.

#### 2.1.2 Reversing of the Hypoxic Microenvironment

Recent studies have indicated that inhibiting histone deacetylase (HDACs) can induce ferroptosis in cells, thereby reversing hypoxia and enhancing the oxidation state. Therefore, designing and synthesizing active SCOPAs targeting HDACs holds promise for addressing the bottlenecks of PDT. Liu et al. proposed a concept of an active‐based photosensitizer that can be used to target HDACs, based on a quinoxalinone scaffold and a pharmacophore migration strategy.^[^ ^69^ ^]^ By combining the key pharmacophores of HDAC inhibitors with the functional groups of SCOPAs, the molecule QpyNHOH was developed. Experiments showed that when QpyNHOH molecules entered the cell nucleus, they could inhibit the activity of HDACs and upregulate histone acetylation. The increased histone acetylation can drive the expression of cancer suppressor genes such as p53 and promote ferroptosis in tumor cells. Subsequently, the oxygen concentration increased, reversing the antioxidant properties. Based on this effect, HDAC‐targeting SCOPAs exhibited more efficient PDT outcomes.

#### 2.1.3 Reducing the Oxygen Dependence

Due to the abnormal blood vessels and excessive proliferation of tumor tissue, the TME is in a state of severe hypoxia (pO_2_ < 5 mm Hg), significantly reducing the clinical efficacy of PDT. Designing nonoxygen‐dependent SCOPAs that can generate ROS under anaerobic conditions is expected to fundamentally solve the oxygen‐dependence problem, but this is an extremely challenging task. Considering the limitations, Zhu's team has synthesized a novel tetravalent platinum photo‐oxidant. This tetravalent platinum photo‐oxidant can oxidize various biomolecules such as hemin, NADH, proteins, and DNA in an oxygen‐independent manner under NIR light irradiation to generate ROS without oxygen, overcoming the limitations of traditional PDT in complex hypoxic TME. Meanwhile, the prodrug itself can be reduced to clinical platinum drugs such as carboplatin or oxaliplatin. Compared to traditional platinum drugs, it had extremely low dark toxicity, and its antitumor activity after activation can exceed that of clinical drugs by up to 461 times, which greatly overcame the resistance of tumor cells to platinum drugs (Figure 1b).^[^ ^70^ ^]^ Additionally, strategies utilizing cationic radicals to directly oxidize water to produce highly cytotoxic •OH, as well as glutathione (GSH)‐driven photochemistry to generate hydrogen radicals (H•) are also promising.^[^ ^71^, ^72^ ^]^

#### 2.1.4 Increasing the Targeting Ability

To further enhance the phototherapeutic effect of type‐II PDT, the development of SCOPAs with specific targeting capabilities is an effective measure to improve treatment outcomes. Guo et al. designed a photosensitizer called TPE‐PyT‐CPS, which can target the Golgi apparatus (GA). Its specific GA targeting ability was achieved through cyanide‐induced molecular rod‐like stacking, and cellular experiments have confirmed that it entered the GA through caveolin/raft‐mediated endocytosis. Upon illumination, it can cause oxidative stress and fragmentation of the GA, thereby activating apoptotic pathways and inhibiting tumors. Compared to non‐GA‐targeted counterparts, TPE‐PyT‐CPS demonstrated superior PDT effects, providing a strategy for developing SCOPAs with specific GA targeting capabilities.^[^ ^73^ ^]^ In addition, SCOPAs targeting other subcellular organelles such as cell membranes,^[^ ^74^ ^]^ mitochondria,^[^ ^75^ ^]^ lysosomes,^[^ ^76^ ^]^ and endoplasmic reticulum have also been developed successively.^[^ ^77^ ^]^ In addition to SCOPAs with inherent targeting functions, carrier targeting is also a commonly used strategy for constructing targeted SCOPAs.^[^ ^78^ ^]^ Liu et al. designed and synthesized an efficient photosensitizer called AIEPS5 with aggregation‐induced near‐infrared emission.^[^ ^79^ ^]^ By linking polyethylene glycol (PEG) to AIEPS5, an amphiphilic polymer called AIEPS5‐PEG2000 was produced, which was self‐assembled into AIEPS5‐NPs in water. By modifying the surface of AIEPS5‐NPs with Her‐2 nanobodies, AIEPS5‐NPs‐NB with precise targeted delivery capabilities can be obtained. Subsequently, they established a patient‐derived xenograft model based on actual patient samples of oral cancer cells and demonstrated that AIEPS5‐NPs‐NB has significantly better treatment outcomes for oral cancer compared to hemoporfin.

#### 2.1.5 Responding to the TME

The overexpression of reducing substances such as GSH in tumor cells can reduce the therapeutic effect of PDT. Liu et al. developed PMOF NPs that accumulate in tumors and release precursors in response to GSH, generating TPATrzPy‐3+ for fluorescence imaging‐guided PDT.^[^ ^80^ ^]^ Wang's group reported DANO, which upon light and GSH exposure, released •NO to form DAPS. DAPS could generate ^1^O_2_ and ONOO‐ through cascade reactions. The selective activation of DANO by GSH minimizes the risk of nonspecific phototoxicity while boosting the cytotoxicity through free radical cascade reactions, thereby enhancing the therapeutic efficacy.^[^ ^81^ ^]^ Abnormal production of intracellular endogenous hydrogen sulfide (H_2_S) is intrinsically linked to diseases such as Alzheimer's disease, cirrhosis, inflammation, and cancer. Based on that, Shi's team had developed an intelligent nano platform called ZNNPs, which could detect and deplete H_2_S, activating PDT for colorectal cancer. This platform monitors H_2_S levels and reduces glycolysis, damaging mitochondria and inhibiting cancer activity (Figure 1c).^[^ ^82^ ^]^ Lin group prepared a series of pH‐activatable SCOPAs (LET‐R, R = H, Cl, Br, I) that can be specifically activated by acidic TME under 808 nm laser excitation to generate ^1^O_2_, achieving TME/light dual‐controllable PDT. Ultimately, complete eradication of 4T1 tumors was achieved using LET‐I‐FA at an ultra‐low laser power density.^[^ ^83^ ^]^

#### 2.1.6 Developing Two‐Photon PDT

Currently, the absorption and emission peaks of most SCOPAs are located in the visible light region. Due to limited penetration ability and strong biological background fluorescence, the diagnosis and treatment effect of deep‐seated tumors is restricted. Therefore, it is crucial to develop PDT with deeper tissue penetration capabilities. Two‐photon photodynamic therapy (TP‐PDT) employs near‐infrared pulsed lasers to excite the SCOPAs, causing it to absorb two NIR photons and generate ROS. The longer excitation light has a deeper penetration depth, exhibiting tremendous potential for treating deep‐seated tumors.^[^ ^84^, ^85^ ^]^ The Li research group designed an assembly based on the natural product emodin (Emo), which possessed superior two‐photon absorption capabilities and was utilized for efficient TP‐PDT. Emo has been characterized as having significant two‐photon absorption properties, capable of generating ^1^O_2_ under near‐infrared femtosecond pulsed laser irradiation, with a ^1^O_2_ quantum yield of 31.9%. After co‐assembly with serum albumin, E/H NPs exhibited enhanced two‐photon absorption and maintained good ^1^O_2_ generation capabilities. The uptake rate of E/H NPs in MCF‐7 cells was higher than that of Emo aggregates, and they demonstrated significant inhibitory effects on MCF‐7 cells under 800 nm femtosecond pulsed laser, confirming their effective TP‐PDT performance.^[^ ^86^ ^]^ Zhang et al. reported a metal‐free nano‐photosensitizer (APDC‐DTPA) based on a thermally activated delayed fluorescence molecule with NIR emission. APDC‐DTPA NPs can be activated by two‐photon excitation to generate ^1^O_2_ for efficient 2P‐PDT, offering better tissue penetration depth and reduced nonspecific tissue damage compared to 1P‐PDT.^[^ ^87^ ^]^ The Tang research group had also developed various two‐photon SCOPAs for 2P‐PDT and achieved a series of excellent performances in the field of tumor diagnosis and treatment.^[^ ^88^, ^89^, ^90^, ^91^ ^]^

#### 2.1.7 Constructing Self‐Luminescence PDT

**Figure 2:**
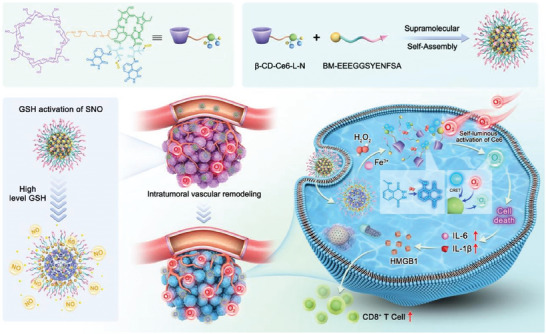
Schematic illustration of tumor microenvironment‐modulating nanoparticles for CRET‐excited photodynamic therapy without external light sources. Reproduced with permission.^[^ ^95^ ^]^ Copyright 2024, Elsevier.

PDT based on self‐luminescence has garnered significant attention from researchers due to its ability to achieve effective optical therapeutic outcomes and its independence from the tissue penetration depth of light.^[^ ^92^, ^93^ ^]^ Building on this concept, Sun et al. developed a tumor microenvironment‐responsive Cherenkov self‐luminescent photosensitizer, ^131^I‐sPS NPs, for deep‐tissue PDT of tumors. This material utilized *N*‐Alpha‐fluorenylmethoxycarbonyl‐*N*‐Epsilon‐*t*‐butoxycarbonyl‐l‐lysine as the linker backbone for the photosensitizer, modified with *N*,*N*‐diisopropylethylenediamine as a pH‐sensitive group, pyropheophorbide a (PPa) as the photosensitizer, ^131^I‐labeled tyrosine as the Cherenkov light donor, and polyethylene glycol hydrophilic long chains to adjust the hydrophilic‐hydrophobic balance of the material. These components self‐assembled to form ^131^I‐sPS NPs. Under neutral initial conditions, ^131^I‐sPS NPs existed in an aggregated state, and their photosensitizing activity was suppressed due to ACQ effect. However, in acidic conditions, ^131^I‐sPS NPs became protonated and disperse, leading to the disappearance of ACQ. Consequently, PPa continuously generated ROS under Cherenkov light illumination, effectively killing tumor cells and ultimately achieving selective eradication of tumor tissues.^[^ ^94^ ^]^ In addition, Jin group has also designed a multifunctional photosensitizer for self‐luminescence PDT through cascaded energy transfer and tumor microenvironment modulation (**Figure** **2**). This multifunctional photosensitizer was synthesized by coupling Ce6, luminol, and the NO donor S‐nitrosoacetylpenicillamine (SNAP) to aminated β‐cyclodextrin (β‐CD‐NH_2_). Through host–host interactions between β‐CD and a benzimidazole (BM)‐covered pancreatic cancer cell‐targeting peptide, it further self‐assembled into supramolecular nanoparticles that target tumors. By triggering chemiluminescence resonance energy transfer (CRET) between luminol and Ce6 via endogenous H_2_O_2_, self‐activated PDT is achieved without the need for an external light source. Due to the higher levels of H_2_O_2_ and Fe^3+^ in cancer cells, the photodynamic effect preferentially eliminated cancer cells with low side effects. Concurrently, the released NO was expected to alleviate tumor hypoxia, reduce intracellular GSH levels, and enhance PDT efficacy.^[^ ^95^ ^]^

### 2.2 Methods to Promote type‐I PDT

Compared with ^1^O_2_, O_2_^•−^ and •OH have higher chemical activity and are more cytotoxic ROS in terms of cellular damage. Furthermore, type‐I PDT has a lower dependence on oxygen compared to type‐II PDT, making it more effective in treating hypoxic regions of tumors. So understanding the design principles of type‐I SCOPAs cannot be ignored.

#### 2.2.1 Reducing T1 State Energy Level to Prevent Oxygen Sensitization

**Figure 3:**
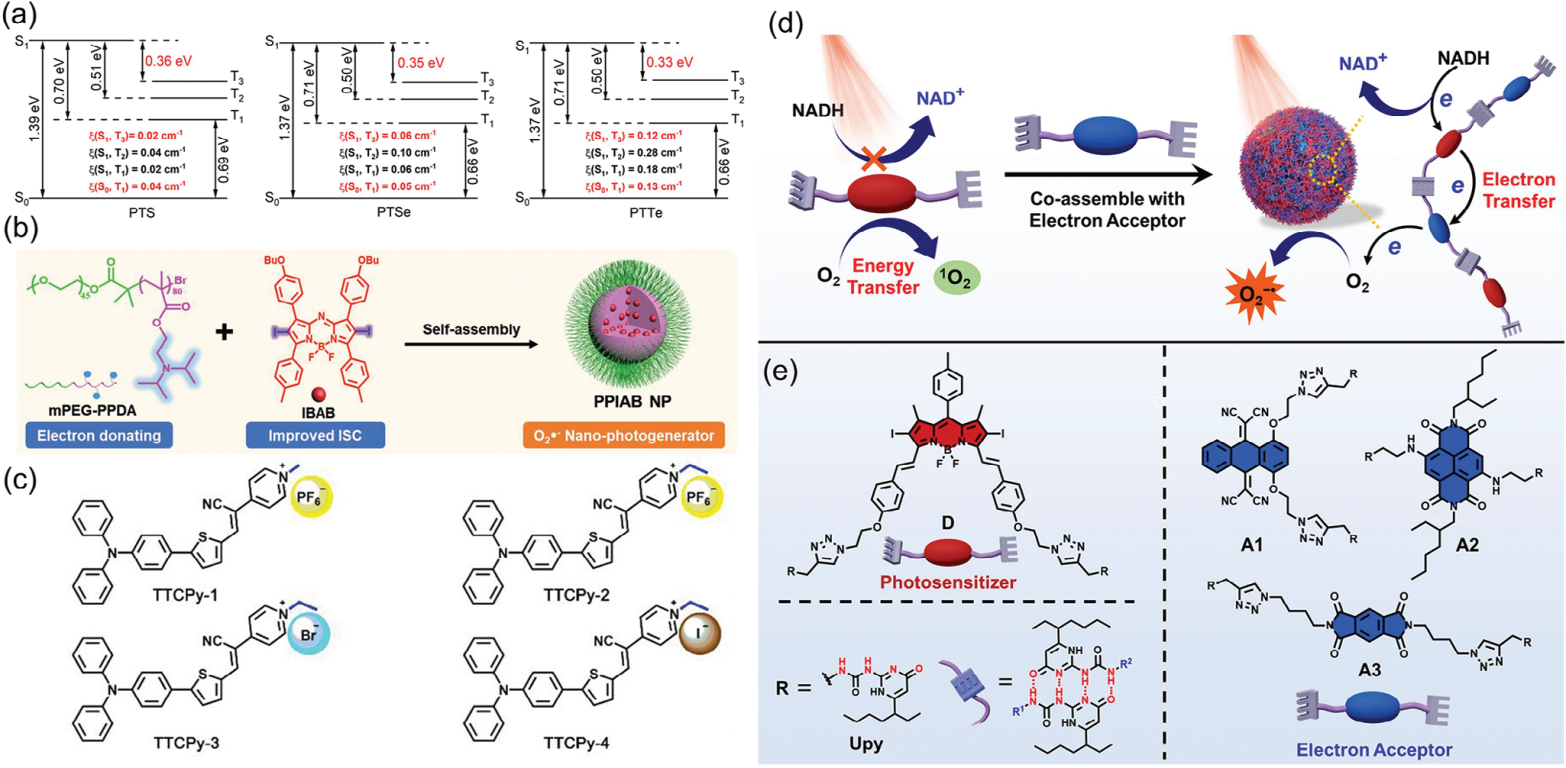
a) Excitation energies of the low‐lying excited states and spin‐orbit coupling constants (ξ(S*_n_*, T*_n_*)) between them based on the optimized S_0_‐geometries of PTS, PTSe, and PTTe oligomers. Reproduced with permission.^[^ ^98^ ^]^ Copyright 2021, Wiley‐VCH. b) Preparation of PPIAB NPs. Reproduced with permission.^[^ ^99^ ^]^ Copyright 2020, Wiley‐VCH. c) Molecular structures of designed type‐I AIE‐PSs. Reproduced with permission.^[^ ^100^ ^]^ Copyright 2022, Wiley‐VCH. d) Schematic illustration of the preparation of the supramolecular photodynamic agent, as well as photoinduced oxidation of NADH and generation of O_2_^•−^. e) Chemical structures of the photosensitizer (D) and electron acceptors (A1, A2, and A3). Reproduced with permission.^[^ ^102^ ^]^ Copyright 2022, Springer Nature.

A breakthrough in inhibiting the energy transfer process is to lower the T_1_ energy of the SCOPAs below the oxygen sensitization threshold of 0.98 eV.^[^ ^96^ ^]^ Yang et al. designed and synthesized BODIPY dimers and trimers that generate O_2_^•−^ only through a type‐I mechanism under illumination, without producing ^1^O_2_. High‐precision theoretical calculations, photophysical and electrochemical studies have shown that the small energy difference between S_1_ and T_2_ states facilitates rapid relaxation of the photosensitizer from T_2_ to T_1_. Additionally, the energy gap between T_1_ and S_0_ is smaller than the energy required to generate ^1^O_2_, thus inhibiting type‐II PDT. Laser flash photolysis experiments demonstrated microsecond‐scale triplet state lifetimes, and electrochemical analysis showed that these molecules have a lower reduction potential, allowing them to sensitize oxygen to generate only O_2_^•−^.^[^ ^97^ ^]^ Similarly, as shown in **Figure** **3**a, Huang's team developed three narrow‐bandgap semiconductor polymers (PTS, PTSe, PTTe) using thiophene isoindigo as the electron acceptor and thiophene, selenophene, and tellurophene as donors. Comparing the HOMO‐LUMO energy levels of the NPs with the redox potential of ROS revealed that the T_1_ state energy was insufficient to sensitize ^3^O_2_ to ^1^O_2_, which facilitated type‐I PDT through intermolecular electron transfer. PTTe NPs exhibited excellent biocompatibility and unprecedented NIR‐II type‐I photodynamic performance under both normoxia and hypoxia.^[^ ^98^ ^]^

#### 2.2.2 Enhancing the Electron Donating‐Accepting or ISC Capabilities

Dong et al. enhanced the electron‐donating ability of the amphiphilic polymer mPEG‐PPDA by introducing an electron‐rich diisopropylamino functional group. In parallel, they improved the ISC capability of the photosensitizer aza‐BODIPY by substituting iodine, leading to the formation of amphiphilic nano‐photosensitizer PPIAB NPs via self‐assembly (Figure 3b). Through effects like disproportionation reaction, Harber–Weiss reaction, and Fenton reaction, PPIAB NPs could effectively generate a charge‐separated state under 660 nm laser irradiation, reacting with oxygen to produce cytotoxic O_2_^•−^. This study provided a novel approach for the rational design of type‐I SCOPAs.^[^ ^99^ ^]^ Additionally, augmenting the electron‐withdrawing ability of the SCOPAs has been also found to promote type‐I ROS generation. The Tang research group creatively introduced a cyano group into the molecular structure of TTPy, and incorporated heavy atoms like Br and I through molecular engineering to develop a series of AIE‐based SCOPAs (Figure 3c). The cyano group acted as an acceptor, strengthening D‐A interactions, while the heavy‐atom effect boosted the ISC process, leading to the generation of the T_1_ state and facilitating type‐I ROS production. The series of SCOPAs demonstrated efficient generation of •OH and O_2_^•−^, enabling broad‐spectrum bacterial imaging‐mediated PDT.^[^ ^100^ ^]^

#### 2.2.3 Increasing Electron Affinity

Phosphorus oxide indole (PIO) derivatives are distinguished by their high stability, low biotoxicity, and membrane permeability, as well as a distinctive electronic structure that grants high electrophilicity. This enables PIO's π‐electron system to effectively attract and stabilize external electrons. Based on this, Zhao et al. incorporated triphenylamine (TPA) and pyridine groups into the molecular structure of PIO to design and synthesize two isomers with AIE characteristics, named α‐TPA‐PIO and β‐TPA‐PIO. Theoretical calculations demonstrated that the efficient ISC efficiency and electrophilicity of β‐TPA‐PIO supported its type‐I ROS generation ability. When exposed to light, β‐TPA‐PIO was found to induce endoplasmic reticulum stress‐mediated apoptosis and autophagy.^[^ ^101^ ^]^

#### 2.2.4 Self‐Assembly Into Supramolecules

In recent years, the supermolecular self‐assembly strategy has become increasingly important in the field of type‐I PDT. A key aspect of designing efficient type‐I PDT involves regulating the pathways of energy transfer and electron transfer. While many reported type‐I PDT have focused primarily on the role of ROS, other photoreaction processes that contribute to tumor damage have often been overlooked. The Yang team introduced a novel preparation strategy for type‐I PDT. They initially developed classic BODIPY‐based type‐II SCOPAs and three different electron acceptor molecules. By utilizing quadruple hydrogen bonding, they co‐assembled the SCOPAs and electron acceptors to create a supermolecular copolymer nano‐photosensitizer.^[^ ^102^ ^]^ The close proximity between the SCOPAs and electron acceptors, along with their matched redox potentials, facilitated the transfer of photoinduced electrons from the SCOPAs to the electron acceptors. These electrons were then transferred to molecular oxygen to produce O_2_^•−^. Furthermore, this process led to the formation of highly oxidizing cationic radicals from the SCOPAs, which effectively oxidize reduced coenzyme I in living cells, intensifying biological damage (Figure 3d‐e).

### 2.3 Design rules for Both Type‐I and Type‐II PDT

A single type of PDT has shown significant success in treating various diseases. However, the limited toxicity of ROS due to low oxygen levels within tumors leaves room for improvement in treatment effectiveness, particularly for type‐II PDT. As a result, scientists have recently turned their attention to SCOPAs capable of generating both type‐I and type‐II ROS simultaneously, with the goal of overcoming hypoxia limitations and maximizing therapeutic outcomes.^[^ ^103^ ^]^ Thanks to the persistent efforts of researchers, some SCOPAs with dual PDT functionality have been gradually identified. Nevertheless, further research is required to delve deeper into this unique structure‐activity relationship from a molecular structural perspective.^[^ ^104^, ^105^, ^106^ ^]^

#### 2.3.1 Biotinylation Strategy

The Song research group serendipitously modified a biotin unit with tumor‐targeting properties into a type‐II photosensitizer, resulting in the development of three SCOPAs capable of utilizing both type‐I and type‐II mechanisms. This innovation effectively reduced the reliance on oxygen in PDT applications. By incorporating the biotin unit, the SCOPAs gained tumor‐targeting abilities while also enhancing O_2_^•−^ generation without compromising the production of ^1^O_2_. Further investigation revealed that biotin served as an electron‐rich substrate within the molecule, and the folded conformation of the molecule facilitated electron transfer between the biotin moiety and the SCOPAs, thereby promoting the type‐I PDT mechanism. Due to variations in structure and photophysical properties among the three SCOPAs, biotinylation emerged as a versatile design strategy for converting type‐II PDT into dual type‐I/II PDT. These SCOPAs exhibited both tumor‐targeting capabilities and resistance to hypoxia.^[^ ^107^ ^]^

#### 2.3.2 Cations Strategy

**Figure 4:**
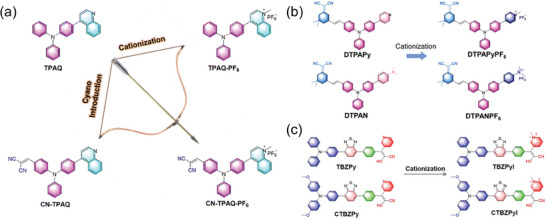
The chemical structures of cationization engineered SCOPAs. a) TPAQ, TPAQ‐PF6, CN‐TPAQ and CN‐TPAQ‐PF6. Reproduced with permission.^[^ ^50^ ^]^ Copyright 2022, The Royal Society of Chemistry. b) DTPAN, DTPAPy, DTPANPF6 and DTPAPyPF6. Reproduced with permission.^[^ ^109^ ^]^ Copyright 2022, Elsevier. c) TBZPy, CTBZPy, TBZPyI and CTBZPyI. Reproduced with permission.^[^ ^110^ ^]^ Copyright 2022, American Chemical Society.

Cationization molecular engineering is a highly effective approach for synthesizing SCOPAs that exhibit both type‐I and type‐II ROS activity.^[^ ^108^ ^]^ Feng et al. selected TPA with a propeller configuration, quinoline, and dicyano as building blocks to synthesize four SCOPAs: TPAQ, TPAQ‐PF6, CN‐TPAQ, and CN‐TPAQ‐PF6 (**Figure** **4**a). Among them, TPA can not only acted as an electron donor but also reduced π‐π stacking in the aggregated state. The incorporation of cyano groups enhanced the light absorption capacity of the molecules, while cationization helped to reduce ΔE_S‐T_, promoted ISC, and enhanced electron transfer ability to facilitate ROS production. Both cationization and cyano groups introduction effectively boosted the production of ^1^O_2_ and •OH. Consequently, CN‐TPAQ‐PF6 exhibited superior ROS‐enhancing capabilities compared to TPAQ, making it a promising candidate for eradicating drug‐resistant bacteria.^[^ ^50^ ^]^ Subsequently, the Feng group expanded their work by developing a series of cation‐engineered organic SCOPAs. As shown in Figure 4b, by employing the cationization strategy, neutral D‐A molecules with a dicyanoisophorone triphenylamine core (DTPAN, DTPAPy) were transformed into cationic A‐D‐A' type SCOPAs (DTPANPF6 and DTPAPyPF6). Experimental and simulation results showed that cationization could promote the ISC process and improve charge transfer and separation, leading to increased generation of •OH and O_2_^•−^.^[^ ^109^ ^]^ They have also developed cationic TBZPyI and CTBZPyI using TBZPy and CTBZPy as neutral precursors, with iodine as the counter anion and pyridinium cationization (Figure 4c).^[^ ^110^ ^]^ After cationization treatment, the cationic portion of CTBZPyI exhibited a stronger electron accepting ability, which effectively promoted electron separation and transfer processes, enhancing the generation of ROS, mainly •OH. Moreover, cationization also conferred upon CTBZPyI the capability to bind to bacteria in the absence of light, resulting in moderated bacterial inactivation. Subsequent light irradiation can further enhance its antibacterial properties.

#### 2.3.3 Modulating Triplet Lifetime and ISC Efficiency

The triplet state lifetime is crucial in controlling the generation of ROS due to the considerable time required for energy and electron transfer. Consequently, in the design of efficient PSs, particularly for generating potent type‐I ROS, numerous studies have focused on pursuing long triplet state lifetimes. Liu et al. synthesized and investigated the properties of the photosensitizer mBDPPyH. Subsequently, the authors modified the structure in two ways: one by further attaching another pyridinium salt as an additional electron acceptor, and the other by introducing bromine to accelerate ISC through the heavy‐atom effect. Th*en*, two additional compounds, mBDP‐2Py and mBDP‐PyBr *were prepared*. Despite similar absorption coefficients, singlet‐triplet energy gaps, and reduction potentials, mBDP‐PyBr exhibited the strongest ROS generation capability for both type‐I and type‐II ROS. Although the heavy‐atom effect of the additional bromine atom resulted in a shortened triplet state lifetime, the significant enhancement of the ISC process from the singlet charge transfer state to the triplet state compensated for this drawback, simultaneously promoting the generation of singlet oxygen and free radicals. Owing to its exceptional ROS generation ability, mBDP‐PyBr NPs were able to induce significant tumor cell death upon illumination, both under normoxic and hypoxic conditions.^[^ ^111^ ^]^

### 2.4 Multimodal Collaborative Treatments Based on PDT

Dealing with complex TME, like hypoxia and overexpressed GSH, using a single PDT mode can be challenging. Utilizing multimodal collaborative treatments based on PDT allow different treatment modes to complement each other, thereby enhancing the treatment effect.

#### 2.4.1 PDT Combined with Chemotherapy

**Figure 5:**
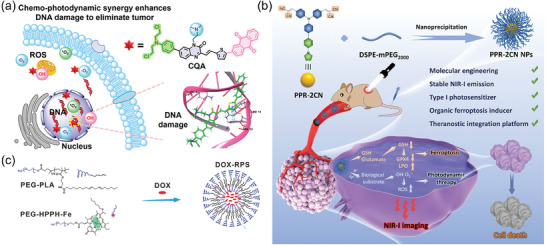
a) Design of the bioactive photosensitizer CQA for PDT and DNA damage. Reproduced with permission.^[^ ^113^ ^]^ Copyright 2024, American Chemical Society. b) Applications of PPR‐2CN NPs for NIR‐I imaging‐guided highly efficient type‐I photodynamic and ferroptosis synergistic therapy. Reproduced with permission.^[^ ^116^ ^]^ Copyright 2023, Wiley‐VCH. c) Schematic illustration of DOX‐RPS for photodynamic‐chemodynamic therapy. Reproduced with permission.^[^ ^121^ ^]^ Copyright 2021, Wiley‐VCH.

PDT can reduce the activity of drug‐resistant proteins and decrease drug efflux, thereby enhancing the sensitivity of chemotherapeutic drugs and further destroying tumor cells when combined with chemotherapy.^[^ ^112^ ^]^ Shi and colleagues recently made a significant advancement by combining DNA‐targeted chemotherapeutic drug moieties with a SCOPA, resulting in synergistic effects for combined chemotherapy and PDT. The DNA alkylating agent, nitrogen mustard, functioned as the chemotherapeutic moiety, effectively targeting DNA by causing crosslinking reactions and inducing DNA damage. The dimethylamine present in the molecule enhanced the interaction between the photosensitizer CQA and DNA while also improving hydrophilicity. Additionally, the introduction of an anthraquinone group with favorable redox properties as an electron acceptor facilitated electron transfer, leading to the production of ROS. Research findings indicated that CQA not only displayed chemotherapy effects through DNA damage but also generated both type‐I and type‐II ROS for PDT, ultimately demonstrating a synergistic effect in chemo‐photodynamic therapy (**Figure** **5**a).^[^ ^113^ ^]^

#### 2.4.2 PDT Combined with Ferroptosis

TME exhibits high redox homeostasis characterized by overexpressed GSH, rendering tumor cells more vulnerable to oxidative stress. GSH, as an antioxidant, shields tumor cells from ROS effects induced by PDT, diminishing treatment efficacy. Due to the pivotal role of GSH in tumors, GSH depletion‐induced ferroptosis has emerged as a promising strategy for multimodal synergistic tumor therapy.^[^ ^114^, ^115^ ^]^ While traditional ferroptosis primarily involves transition metal ions and GSH, the potential biological toxicity of transition metals poses a risk. Limited literature exists on the use of nonmetallic SCOPAs to directly induce ferroptosis in cells. Our research team has developed a novel series of SCOPAs for combined ferroptosis and PDT treatments in tumors, with the photosensitizer PPR‐2CN, featuring an A‐D‐A structure, demonstrating superior performance. Upon irradiation, PPR‐2CN could generate significant ROS levels and interact directly with glutamate and GSH, inhibiting GSH synthesis in cells, promoting redox imbalance, and facilitating ROS‐mediated ferroptosis. Biological experiments have confirmed the outstanding antitumor efficacy and minimal side effects of PPR‐2CN NPs. This study represented a successful implementation of SCOPAs that functioned as both a type‐I photodynamic agent and a nonmetallic ferroptosis inducer for multimodal collaborative tumor diagnosis and treatment (Figure 5b).^[^ ^116^ ^]^

#### 2.4.3 PDT Combined with Chemodynamic Therapy

Chemodynamic therapy (CDT) mainly relies on the TME to trigger the Fenton (or Fenton‐like) reaction within nano‐drugs, producing highly reactive •OH radicals for targeted tumor therapy. This innovative approach has attracted significant global interest in the medical field since it was proposed by Academician Shi's team in 2016.^[^ ^117^, ^118^, ^119^, ^120^ ^]^ Chen et al. developed doxorubicin‐loaded ROS‐responsive polymeric vesicles (DOX‐RPS) by self‐assembling amphiphilic polyethylene glycol‐polylinoleic acid and PEG‐HPPH‐Fe (Figure 5c). These vesicles effectively transported drugs to tumors via passive targeting. When exposed to laser, the photosensitizer HPPH generateed ROS, leading to oxidation of the linoleic acid chain within the vesicles and subsequent drug release. Interestingly, HPPH‐Fe catalyzed the regeneration of ROS from linoleic acid peroxide through a chemodynamic process. This unique mechanism enabled drug release triggered by ROS without excessive consumption. Both in vitro and in vivo studies confirmed that the production of ROS initiated drug release and enhanced the anti‐tumor efficacy of DOX‐RPS. The photodynamic‐chemodynamic cascade strategy presents a promising approach for improving combination therapy.^[^ ^121^ ^]^

#### 2.4.4 PDT Induced Immunogenic Cell Death

Immunogenic cell death (ICD) is a specialized form of apoptosis that enhances the immunogenicity of tumor cells through the release of damage‐associated molecular signals. This activation prompts antigen‐presenting cells to more efficiently recognize and target tumor antigens, thereby initiating a specific anti‐tumor immune response.^[^ ^122^, ^123^ ^]^ Currently, only a limited number of chemotherapy drugs, such as methotrexate, doxorubicin, and oxaliplatin, have been shown to effectively induce ICD. Additionally, only a few SCOPAs have been demonstrated to trigger ICD by inducing oxidative stress in tumor cells.

**Figure 6:**
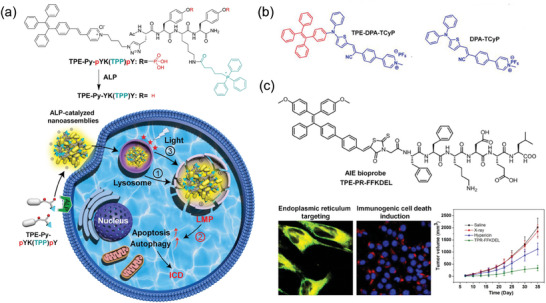
SCOPAs targeting organelles for PDT‐induced ICD. a) Structure of the photosensitizer targeting lysosomes. Reproduced with permission.^[^ ^124^ ^]^ Copyright 2021, Wiley‐VCH. b) Structure of the photosensitizer targeting mitochondria. Reproduced with permission.^[^ ^125^ ^]^ Copyright 2019, Wiley‐VCH. c) Structure of the photosensitizer targeting endoplasmic reticulum. Reproduced with permission.^[^ ^126^ ^]^ Copyright 2020, Springer Nature.

Given that, the Ding research group has recently made significant breakthroughs. They have successfully developed a series of SCOPAs that can precisely target subcellular organelles and significantly enhance the ICD level of tumor cells through PDT. These achievements specifically include: 1) Lysosome‐targeted PDT‐induced ICD: The Ding research group reported an innovative lysosome membrane permeabilization (LMP) inducer called TPE‐Py‐pYK(TPP)pY (**Figure** **6**a). This inducer can accumulated in the lysosomes of cancer cells overexpressing alkaline phosphatase (ALP) and respond to ALP, leading to the formation of nano‐assemblies and the generation of ^1^O_2_. Through this process, TPE‐Py‐pYK(TPP)pY could effectively induce LMP and lysosome membrane rupture, triggering significant ICD and successfully converting the originally immune‐“cold” tumor into “hot” tumor, promoting the infiltration of a large number of CD8^+^ and CD4^+^ T cells into the tumor tissue.^[^ ^124^ ^]^ 2) Mitochondria‐targeted PDT‐induced ICD: Ding et al. synthesized two novel mitochondrial‐targeted SCOPAs, TPE‐DPA‐TCyP and DPA‐TcyP, with a unique twisted 3D structure. These two SCOPAs were found to enhance ICD by inducing oxidative stress in cancer cell mitochondria. Experimental results demonstrated that TPE‐DPA‐TCyP effectively targeted mitochondria, leading to mitochondrial oxidative stress, increased endoplasmic reticulum CRT translocation, and the release of adenosine triphosphate (ATP), high mobility group box 1 protein (HMGB1), and HSP 70. This process significantly boosted the ICD of cancer cells (Figure 6b).^[^ ^125^ ^]^ 3) Endoplasmic reticulum‐targeted PDT‐induced ICD: The Ding team further developed a biological probe named TPE‐PR‐FFKDEL, which covalently links a photosensitizer molecule with the endoplasmic reticulum‐targeting peptide FFKDEL, achieving efficient ICD induction in tumor cells. Due to its abundant motion units, this probe still exhibited strong fluorescence emission and ^1^O_2_ generation ability in the aggregated state. Cellular experiments have shown that TPE‐PR‐FFKDEL, in comparison to the commonly used hypericin, could trigger the release of more immunostimulatory damage‐associated molecules.(Figure 6c).^[^ ^126^ ^]^ These significant research findings not only offered new insights for creating effective immunogenic cell death inducers, but also broaden the potential applications of SCOPAs in PDT‐mediated immunotherapy. The above applications of PDT mediated by SCOPAs have been concluded in **Table** **1**.

**Table 1:** The applications of PDT mediated by SCOPAs.

Name	Abs^a)^ [nm]	Laser [nm]	Power [W cm^−2^]	ROS type	Strategy	Ref.
C3T‐Pc	662	655	0.30	^1^O_2_	2.1.1	[66]
TPETCAQ	520	594	0.30	^1^O_2_	2.1.1	[68]
QpyNHOH	425	450	0.10	^1^O_2_	2.1.2	[69]
DA	600	White light	0.04	•OH	2.1.3	[71]
TAF	365	800	0.05	H•	2.1.3	[72]
TPE‐PyT‐CPS	487	532	0.01	^1^O_2_	2.1.4	[73]
AIEPS5‐NPs‐NB	475	532	0.10	^1^O_2_	2.1.4	[79]
PMOF	/	400–700	0.10	^1^O_2_	2.1.5.1	[80]
DANO	405	405	0.02	^1^O_2_	2.1.5.1	[81]
ZNNPs	650	660	0.05	^1^O_2_	2.1.5.2	[82]
DBP‐SPy	800	808	0.2	^1^O_2_	2.1.5.3	[83]
E/H	400	800	3.00	^1^O_2_	2.1.6	[86]
NIR‐TADF	525	880	1.00	^1^O_2_	2.1.6	[87]
1a	628	400–800	0.02	O_2_^•−^	2.2.1	[97]
PTTe	970	1064	1.00	O_2_^•−^	2.2.1	[98]
PPIAB	660	660	0.05	O_2_^•−^	2.2.2	[99]
TTCPy‐3	534	White light	0.016	O_2_^•−^ •OH	2.2.2	[100]
β‐TPA‐PIO	563	White light	0.02	•OH	2.2.3	[101]
DA1	665	660	0.02	O_2_^•−^	2.2.4	[102]
Compound 1	400‐700	White light	0.02	O_2_^•−^, ^1^O_2_	2.3.1	[107]
Pys‐QM‐TT	477	White light	0.08	^1^O_2_, O_2_^•−^, •OH	2.3.2	[108]
DTPANPF_6_, DTPAPyPF_6_	498, 480	White light	0.02,	^1^O_2_, O_2_^•−^, •OH,	2.3.2	[109]
CTBZPyI	500–700	White light	0.02	•OH, ^1^O_2_	2.3.2	[110]
CQA	450	Green light	0.10	^1^O_2_, •OH, O_2_^•−^	2.4.1	[113]
PPR‐2CN	484	White light	0.015	•OH, O_2_^•−^	2.4.2	[116]
DOX‐RPS	670	671	0.10	^1^O_2_	2.4.3	[121]
TPE‐Py‐pYK(TPP)pY	396	White light	0.25	^1^O_2_ •OH	2.4.4	[124]
TPE‐DPA‐TCyP	504	White light	0.01	^1^O_2_	2.4.4	[125]
TPE‐PR‐FFKDEL	430	White light	0.25	/	2.4.4	[126]

^a)^ The absorption wavelength of SCOPAs.

## 3 Photothermal Therapy

PTT is a tumor treatment approach that involves the use of photothermal reagents to convert light energy into heat energy when exposed to laser irradiation. This process induces local hyperthermia, leading to the destruction of tumor cells. Due to its noninvasive, long‐lasting, and safe characteristics in tumor treatment, it has become a hot topic in the research of new cancer treatment methods. PTT based on nanomaterials has been widely used in clinical treatment and laboratory scientific research.

### 3.1 Approaches to Boost NIR‐I Photothermal Conversion

Compared to visible light, NIR light with a longer wavelength has a greater penetration depth into biological tissues and is referred to as the “biological optical window.” This characteristic has garnered significant interest in the biomedical field.^[^ ^127^ ^]^ Nevertheless, it is essential to continue developing strategies that enhance NIR‐I photothermal conversion to further progress PTT.

#### 3.1.1 Facilitating Intramolecular Bond Stretching Vibration

Currently, the main avenues to promote photothermal conversion involve regulating photoinduced electron transfer, fluorescence resonance energy transfer between fluorescent molecules and quenchers, and their own π‐π stacking effects. However, these approaches necessitate the introduction of control factors such as quenchers, intermolecular stacking, and intermolecular cavities, making it more complex to material design. Ding et al. introduced a novel concept of intramolecular bond stretching vibration‐induced photothermal conversion, highlighting its spontaneity, intensity, and independence from external factors of chemical bond stretching vibrations.^[^ ^128^ ^]^ The researchers synthesized electron donor‐acceptor compounds DCP‐TPA and DCP‐PTPA with pyrazine electron acceptor units. Despite exhibiting AIE behaviors, these molecules showed remarkably low fluorescence quantum efficiency in their aggregated and crystalline states, contrary to the common belief that AIE molecules emit strongly in the crystalline state due to restricted molecular motion. Theoretical calculations indicated that the stretching vibration of the C‐N bond played a crucial role in excited state energy dissipation in both photosensitizer molecules, thereby promoting photothermal conversion. DCP‐TPA NPs and DCP‐PTPA NPs demonstrated impressive PCE of 52% and 59% under 660 nm laser, respectively.

#### 3.1.2 Providing Space for Molecular Motion

The introduction of AIE units with strong D‐A structure and propeller configurations into the molecular backbone can enhance intramolecular motion, thereby improving photothermal conversion. However, in the aggregated state, intramolecular motion is often hindered. To tackle this issue and boost the nonradiative transition capability of molecules in the aggregated state, Ding's team proposed a novel molecular strategy involving the incorporation of long alkyl branched side chains. This innovative design mitigated the impact of intermolecular interactions, created space for molecular movement, and promoted the free rotation of AIE motifs. As a result, this approach activated molecular motion in the aggregated state and enhanced the nonradiative transition capabilities.^[^ ^129^, ^130^ ^]^

**Figure 7:**
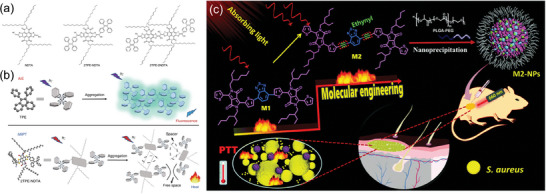
a) Molecular structures of NDTA, 2TPE‐NDTA, and 2TPE‐2NDTA. b) Schematic diagram of the working mechanism of AIE and iMIPT. Reproduced with permission.^[^ ^129^ ^]^ Copyright 2019, Springer Nature. c) The molecular engineering to enhance the photothermal performance by the introduction of ethynyl. Reproduced with permission.^[^ ^131^ ^]^ Copyright 2021, The Royal Society of Chemistry.

The team designed three molecules, NDTA, 2TPE‐NDTA, and 2TPE‐2NDTA, for validation (**Figure** **7**a). The latter two, containing the TPE rotor unit, showed fluorescence quenching in solution due to active molecular rotation, while emitting strong fluorescence at low temperatures in the solid state by restricting molecular motion. Ultrafast spectroscopy experiments indicated that the introduction of TPE led to dominant nonradiative transitions of excitons, supporting the rationale and effectiveness of incorporating long alkyl side chains in “intramolecular motion‐induced photothermal conversion (iMIPT)” type molecules. Solid‐state NMR tests revealed that 2TPE‐NDTA and 2TPE‐2NDTA had very short relaxation times, suggesting fast molecular motion in the solid state, consistent with their nonluminescent characteristics in that state (Figure 7b). After clarifying the working mechanism, the authors prepared the small molecules into NPs, which exhibited a high PCE of up to 54.9%.

#### 3.1.3 Enlarging the Reservoir

Currently, much research focuses on promoting photothermal conversion by enhancing the nonradiative transitions of photothermal materials. In fact, improving the light absorption capacity of materials can also boost their photothermal performance. The Zhang’ group utilized molecular engineering techniques to introduce ethynyl groups into the conjugated system of thiophene and benzodiazole, which significantly improved the molecule's light absorption ability and enhanced photothermal effects at lower doses (Figure 7c). Their study showed that M2‐NPs had a much higher light absorption efficiency in the 600–700 nm range compared to M1‐NPs, with a mass extinction coefficient approximately twice as high. Furthermore, neither type of NPs exhibited a fluorescence emission peak, indicating that M2‐NPs, with their higher mass extinction coefficient, could absorb more light to generate heat. This study offered a novel approach to enhancing the photothermal performance of SCOPAs.^[^ ^131^ ^]^ Similarly, Li et al. incorporated a twisted structure composed of benzobis(thiadiazole)‐alkylthiophene and a bulky TPA molecular rotor into a planar conjugated diketopyrrolopyrrole unit. The resulting material exhibited a molar absorption coefficient of 2.1×10^5^ L mol^−1^ cm^−1^ at 808 nm and a PCE of 60.4%, successfully achieving NIR‐II fluorescence‐guided surgical navigation and tumor PTT.^[^ ^132^ ^]^

#### 3.1.4 Self‐Assembly‐Induced Crystallization

Crystallization is an effective method to maximize the packing density of molecules, which often leads to highly frequent intermolecular collisions to quench fluorescence and inhibit ISC, thereby enhancing the photothermal performance of SCOPAs. Li et al. reported a strategy to achieve self‐assembly‐induced crystallization (SAIC) in a physiological environment, which can improve the PCE of SCOPAs.^[^ ^133^ ^]^ Specifically, the authors rationally designed three novel heptamethine cyanine‐derived SCOPAs named Cy7‐TCF‐EMBI, Cy7‐TCF‐ETBI, and Cy7‐TCF‐DTX. Cy7‐TCF‐ETBI and Cy7‐TCF‐EMBI, which have limited conformational freedom, could form nanocrystals in water without the help of stabilizers and excipients, exhibiting an SAIC phenomenon that greatly promoted photothermal conversion. Cy7‐TCF‐EMBI had a higher crystallinity and tighter stacking, further increasing the possibility of nonradiative relaxation generating heat. In contrast, Cy7‐TCF‐DTX, which had a stronger D‐A effect but higher molecular freedom, only formed amorphous aggregates and exhibited poor photothermal phenomena in aqueous solutions. In summary, the strategy through SAIC provided a conceptually practical and simple method for boosting photothermal conversion.

### 3.2 Principles for Constructing NIR‐II SCOPAs

Most SCOPAs reported to date have excitation wavelengths in the red (630–700 nm) or NIR‐I (700–950 nm) ranges, making clinical translation difficult due to their limited penetration depth. In contrast, NIR‐II (1000–1700 nm) offers greater tissue penetration depth because of lower light scattering in biological tissues.^[^ ^134^, ^135^, ^136^ ^]^ Additionally, according to the American National Standards Institute's (ANSI) laser safety standard (Z136.1‐2000), the maximum permissible exposure intensity for skin is 0.2 W cm^−2^ at 660 nm, 0.33 W cm^−2^ at 808 nm, and 1 W cm^−2^ at 1000–1100 nm. Therefore, developing NIR‐II SCOPAs with a maximum absorption wavelength exceed 1000 nm can not only enhance PCE and therapeutic outcomes but also reduce damage to normal tissues, making them highly valuable in biomedical applications. However, constructing high‐performance SCOPAs in the NIR‐II window still remains a significant hurdle for the further development and application of organic photothermal agents.

#### 3.2.1 Enhancing D‐A Strength

**Figure 8:**
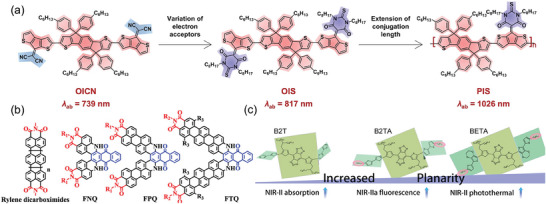
Principles for constructing NIR‐II SCOPAs. a) Tuning the band gap via variation of electron acceptors and extension of conjugation length. Reproduced with permission.^[^ ^140^ ^]^ Copyright 2022, Wiley‐VCH. b) Molecular structure of rylene dicarboximides, FNQ, FPQ, and FTQ. Reproduced with permission.^[^ ^142^ ^]^ Copyright 2023, American Chemical Society. c) Schematic of the design principle of NIR‐II‐absorbing conjugated small molecules by Improving molecular flatness. Reproduced with permission.^[^ ^143^ ^]^ Copyright 2023, Wiley‐VCH.

Introducing electron donor‐acceptor units to create a push‐pull structure and extending the conjugation of molecules are common strategies used in the construction of long‐wavelength absorbing SCOPAs.^[^ ^137^, ^138^, ^139^ ^]^ The Wang’ group modified the band gap of conjugated polymers by incorporating a quinone‐type donor‐acceptor structure and enhancing intramolecular noncovalent interactions. Initially, they developed two oligomers, OICN and OIS, each with distinct quinone structures but the same donor unit (**Figure** **8**a). Energy level calculations and spatial simulations confirmed that OIS had a larger conjugated structure and unique noncovalent “conformational lock” features. To further narrow the band gap, the researchers increased the conjugation length of the molecule through polymerization, resulting in a conjugated polymer, PIS, with strong absorption in the NIR‐II range. In vitro experiments demonstrated that the PIS NPs solution displayed notable photothermal conversion capability and effectively eliminated tumor cells upon exposure to 1064 nm laser irradiation. This research served as a valuable reference for designing NIR‐II SCOPAs.^[^ ^140^ ^]^

#### 3.2.2 Extending π Conjugation Degree

The Yin team presented a novel perylene‐imide‐based NIR‐II SCOPAs for the precise diagnosis and treatment of gliomas. By incorporating a strong electron donor, diaminoanthraquinone, into two perylene monoimide units using a dual modification approach, a D‐A structure was constructed. Subsequently, they extended the degree of π conjugation through a ring‐closing reaction, synthesizing a NIR‐II absorbing SCOPAs (N1). The authors then utilized an amphiphilic functional polymer of 2‐methacryloyloxyethyl phosphorylcholine to encapsulate N1, forming N1@2P NPs capable of crossing the blood‐brain barrier via receptor‐mediated transport. These NPs demonstrated significant blood‐brain barrier permeability, enabling NIR‐II photoacoustic imaging and PTT for deep in‐situ gliomas.^[^ ^141^ ^]^ Additionally, the team conducted C‐N coupling between amino‐substituted terrylenedicarboximide (TMI) and 1, 4‐dichloroanthraquinone, followed by base‐promoted dehydrogenation cyclization to produce a terrylene‐anthraquinone dimer (FTQ) with NIR‐II absorption. FTQ, with an extended π conjugated system, exhibited an absorption peak at 1140 nm extending to the 1500 nm range (Figure 8b). FTQ NPs achieved a 49% PCE under 1064 nm laser irradiation, while maintaining good biocompatibility, photostability, and photoacoustic imaging performance. Both in vitro and in vivo experimental results highlighted the remarkable potential of FTQ NPs‐based photoacoustic imaging‐guided NIR‐II PTT in the diagnosis and treatment of in situ liver cancer.^[^ ^142^ ^]^

#### 3.2.3 Improving Molecular Flatness

Significant progress has been made in research focusing on design strategies such as conjugation extension to enhance the absorption range of D‐A‐D type SCOPAs for improved NIR‐II absorption and enhanced phototherapeutic performance. Molecules with rigid planar structures, as opposed to traditional twisted molecules, often exhibit a high molar absorption coefficient due to limited free bond vibration/rotation, thereby expanding the upper limit of the output signal. Molecular planarity appears to be crucial in the development of optimal NIR‐II SCOPAs. Sun et al. conducted a study where they designed a range of SCOPAs with varying degrees of molecular planarity through D‐A‐D/side‐chain engineering (Figure 8c). The absorption range of SCOPAs shifted toward the NIR‐II region with an increase in the number of thiophenes. However, an excessive number of thiophenes could potentially reduce brightness and photothermal effects in the NIR‐IIa (1300‐1400 nm) range. Research indicated that introducing terminal nonconjugated alkyl chains could enhance the absorption coefficient of SCOPAs in the NIR‐II region, leading to improved fluorescence brightness and photothermal effects in NIR‐IIa. Femtosecond transient absorption spectroscopy revealed a short‐lived intermediate that depleted most of the excited state through nonradiative decay. The formation of this femtosecond intermediate was attributed to the molecular planarity resulting from the combined influence of the number of thiophenes and the terminal alkyl chains. Furthermore, a SCOPA crafted by replacing the exposed thiophene groups with 3, 4‐ethylenedioxythiophene‐2‐one demonstrated high absorption at 1064 nm and exhibited a high NIR‐II PCE, making it suitable for advanced NIR‐II phototherapy in vivo.^[^ ^143^ ^]^

#### 3.2.4 Developing Organic Conjugated Polymers

**Figure 9:**
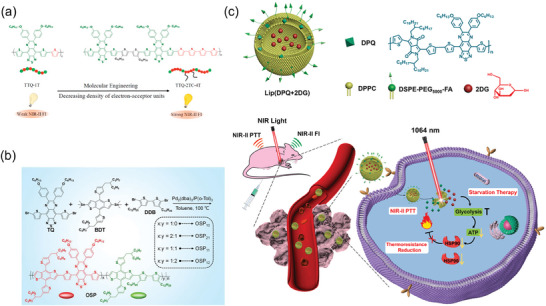
a) Schematic diagram of the molecular mechanisms of the conjugated polymers with enhancement of NIR‐II fluorescence by electron acceptor density adjust effect.^[^ ^145^ ^]^ Copyright 2022, Elsevier. b) Synthetic routes of OSPs with different DDB doping rates.^[^ ^146^ ^]^ Copyright 2021, Wiley‐VCH. c) Synthesis of the lip (DPQ + 2DG) nano‐platform and NIR‐II photo‐triggered starvation therapy assisted PTT. Reproduced with permission.^[^ ^147^ ^]^ Copyright 2021, Elsevier.

In addition to organic small molecule, organic conjugated polymers with excellent optical properties and low biological toxicity have also demonstrated promising potential for NIR‐II PTT.^[^ ^144^ ^]^ Based on the background, Fan's team has developed a series of conjugated polymers for NIR‐II fluorescence imaging and 1064 nm laser‐excited NIR‐II PTT. Firstly, they utilized quaterthiophene (4T) and bithiophene (2TC) with long alkyl side chains to reduce electron‐acceptor density in the main chain of polymers. The NIR‐II fluorescence intensity of the synthesized 1064 nm absorbing polymer TTQ‐2TC‐4T in toluene solution is about 7.30 times higher than that of the undoped TTQ‐1T. Additionally, they constructed TTQ‐MnCO NPs based on TTQ‐2TC‐4T and CO donor (Mn_2_(CO)_10_) for NIR‐II fluorescence imaging‐guided 1064 nm laser‐triggered NIR‐II photothermal/gas synergistic therapy. (**Figure** **9**a).^[^ ^145^ ^]^ Subsequently, by replacing weak electron donor TC with 5, 5'‐dibromo‐4, 4'‐didodecyl‐2, 2'‐bithiophene (DDB), self‐luminous polymers OSP21, OSP11, and OSP12 with gradient NIR‐II brightness were synthesized (Figure 9b). These polymers exhibited distinct NIR‐II emission peaks around 1044 nm. OSPN12 showed promising PTT performance in HepG2 cells under NIR‐II laser irradiation. When the concentration of OSPN12 reached 0.2 mg mL^−1^, the cell survival rate was only 13%.^[^ ^146^ ^]^ Furthermore, researchers successfully synthesized a novel nanoplatform by encapsulating the semiconductor polymer (DPQ) and the glycolysis inhibitor 2‐deoxy‐glucose (2DG) into folate‐modified liposomes (Figure 9c). This platform enabled NIR‐II fluorescence imaging by specifically targeting folate receptors and induced outstanding NIR‐II photothermal effect for the controlled release of 2DG. The release of 2DG inhibited tumor anaerobic glycolysis, cutting off the energy supply to tumor cells and reducing HSP production by lowering ATP levels, which enhanced tumor sensitivity to PTT and demonstrated excellent therapeutic efficiency.^[^ ^147^ ^]^

#### 3.2.5 Constructing Charge Transfer Complexes

Numerous strategies have been devised for creating NIR‐II SCOPAs, but the intricate design and laborious synthesis process have impeded the progress of new SCOPAs. Hence, streamlining the synthesis pathway is crucial to address the increasing need. Charge transfer complexes (CTC) entail substantial charge transfer from an electron donor to an acceptor, leading to electron delocalization across the two molecules. By adjusting the energy gap of CTCs, their capability in light harvesting and amplification, along with bioimaging applications, has been validated.^[^ ^148^, ^149^ ^]^

**Figure 10:**
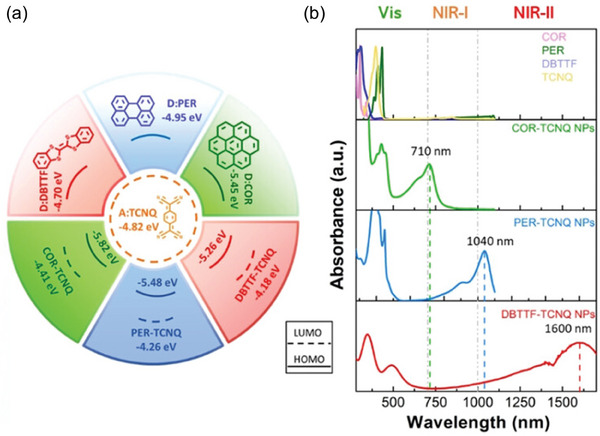
a) Calculated energy diagrams and their corresponding donors and acceptors. b) Absorption spectra of three CT NPs aqueous dispersion and their corresponding monomers dissolved in THF. Reproduced with permission.^[^ ^150^ ^]^ Copyright 2021, Wiley‐VCH.

The energy gap of CTCs is determined by the highest occupied molecular orbital (HOMO) energy level of the donor and the lowest unoccupied molecular orbital (LUMO) energy level of the acceptor. The absorption properties of CTCs can be easily tailored by adjusting their components. The Lee’ group synthesized a variety of CT NPs using different common donor and acceptor molecules, as illustrated in **Figure** **10**. These CT NPs exhibited absorption peaks that can be fine‐tuned from the NIR‐I region to the NIR‐II region, resulting in a range of CT NPs with distinct NIR responses. In particular, CT NPs formed by perylene and 7, 7, 8, 8‐tetracyanoquinodimethane displayed a noticeable red‐shifted absorption peak at 1040 nm. When excited with a 1064 nm laser, the temperature of the aqueous solution containing these CT NPs increased rapidly by 34 °C in just 12 min, achieving a PCE of 42%. Experimental results have demonstrated the effectiveness of CT NPs against both gram‐negative and gram‐positive bacteria.^[^ ^150^ ^]^

### 3.3 Ideas for Designing SCOPAs with Ultrahigh PCE

PCE is a crucial factor in assessing the photothermal efficacy of SCOPAs. The level of PCE is directly linked to the laser intensity needed for PTT. Since high‐intensity lasers can potentially harm normal skin and tissue, enhancing the PCE of photothermal agents and minimizing laser intensity are pivotal challenges in PTT area. Nevertheless, there is currently no standardized protocol for developing SCOPAs with ultrahigh PCE. Therefore, it is imperative to summarize and study various strategies that can promote efficient photothermal conversion.

#### 3.3.1 H‐Aggregation

**Figure 11:**
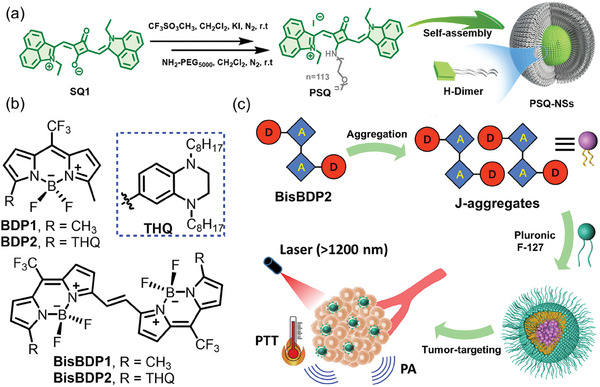
a) Synthetic route from SQ1 to the target product, PSQ, and schematic illustration for PSQ self‐assembly in aqueous solution. Reproduced with permission.^[^ ^151^ ^]^ Copyright 2022, Wiley‐VCH. b) Chemical structures of BDP1, BDP2, BisBDP1, and BisBDP2. c) Schematic illustration of the PA imaging‐guided PTT in the NIR‐II window using BisBDP2 J‐aggregates. Reproduced with permission.^[^ ^155^ ^]^ Copyright 2022, American Association for the Advancement of Science (AAAS).

Molecular H‐aggregates typically exhibit a face‐to‐face stacked arrangement with significant overlap of π‐orbitals, leading to effective quenching of intrinsic fluorescence through intermolecular energy‐trapping exciton interactions, thereby enhancing heat generation via nonradiative energy dissipation. However, the literature on H‐aggregated SCOPAs has been relatively limited thus far, and achieving well‐organized and stable H‐molecular aggregates remains a significant challenge. Wang et al. synthesized an amphiphilic squaraine dye named PSQ, which could spontaneously assemble in aqueous solution to form uniformly sized nanospheres referred to as PSQ‐NSs, featuring a distinctive H‐dimeric substructure (**Figure** **11**a).^[^ ^151^ ^]^ Molecular dynamics simulations provided insights into the self‐assembly process. The low‐frequency out‐of‐plane vibrational modes of the H‐dimer PSQ accelerated nonradiative decay, while the H‐dimer nanospheres demonstrated significantly reduced fluorescence and ^1^O_2_ generation, converting absorbed photons into heat. Consequently, under laser excitation (0.3 W cm^−2^ at 808 nm), the PCE of PSQ‐NSs could achieve an exceptionally high value of 81.2%. In in vivo PTT, PSQ‐NSs exhibited notable characteristics such as high water solubility, physiological stability, and biocompatibility. Injection of PSQ‐NSs intravenously, followed by laser irradiation, led to substantial tumor ablation in mice. Thus, the self‐assembly of H‐dimers offered a novel strategy for the precise design of SCOPAs with ultrahigh PCE.

#### 3.3.2 J‐aggregation

J‐aggregates are characterized by their significant red‐shifted absorption band that occurs after J‐coupling in the excited state, providing an exciting opportunity to shift the NIR‐I absorption of SCOPAs into the NIR‐II window. Researchers have successfully developed J‐aggregates with NIR‐II absorption using cyanine and squaraine.^[^ ^152^, ^153^, ^154^ ^]^ BODIPY dyes, a well‐known classic dye, are considered as parallel J‐aggregated scaffolds alongside cyanine, squaraine, diimide, and chlorophyll dyes. Liu et al. designed a vinyl‐bridged BODIPY scaffold with strong J‐aggregation ability, revealing that the bridged vinyl unit was key to intermolecular J‐coupling regulation (Figure 11b). By incorporating electron‐donating groups into the scaffold, the researchers created a BODIPY dye called BisBDP2, which exhibited a J‐aggregate absorption peak at around 1300 nm (Figure 11c). The BisBDP2 J‐aggregate showed impressive photothermal performance, including a robust photoacoustic response and a PCE of up to 63%. in vivo experiments demonstrated the potential of J‐aggregates in applications such as photoacoustic imaging and PTT for deep‐seated tumors.^[^ ^155^ ^]^

#### 3.3.3 Barrier‐Free Rotation

**Figure 12:**
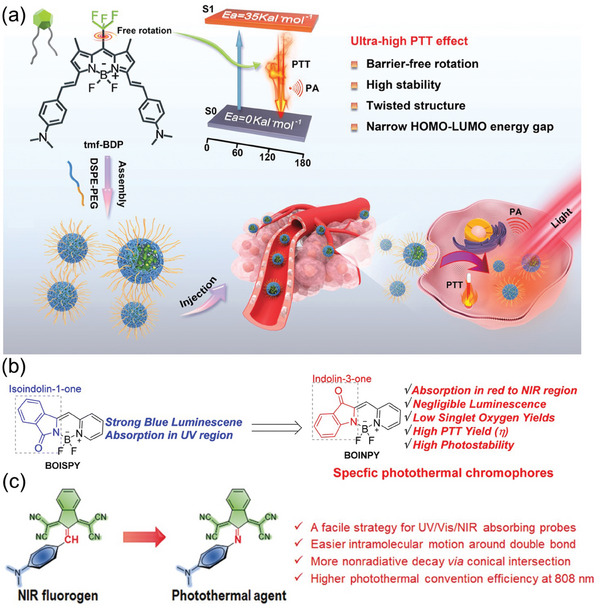
a) Molecular structure of tfm‐BDP. NIR light absorption can relax via barrier‐free rotation of the –CF3 group to generate heat. X axis indicates the rotation angle of –CF3 group. Schematic illustration of self‐assembly and PTT guided by PA imaging using tfm‐BDP NPs. Reproduced with permission.^[^ ^156^ ^]^ Copyright 2020, Wiley‐VCH. b) The structures of BOISPY and BOINPY. Reproduced with permission.^[^ ^158^ ^]^ Copyright 2023, The Royal Society of Chemistry. c) Design strategy of efficient photothermal agent based on double bond molecular motor. Reproduced with permission.^[^ ^159^ ^]^ Copyright 2020, Wiley‐VCH.

The Peng’ group developed a SCOPA tfm‐BDP by incorporating strong electron‐withdrawing groups (–CF_3_) and strong electron‐donating groups (*N*,*N*‐dimethylamino) to establish a robust D‐A interaction. tfm‐BDP demonstrated extensive conjugation, a lengthy absorption wavelength, a high absorption coefficient, and impressive photothermal properties. When exposed to 808 nm laser, the unrestricted rotation of the CF_3_ group enabled tfm‐BDP to primarily return to its ground state through nonradiative transitions, effectively converting light energy into thermal energy. Its twisted molecular configuration prevented π–π stacking and allowed nanoparticles to freely rotate in an aggregated state, resulting in a notable PCE of 88.3% (**Figure** **12**a).^[^ ^156^ ^]^ Dong et al. synthesized four D‐A polyarylpyrrole molecules, namely MAP1‐FE to MAP4‐FE, revealing that different nitrogen‐containing donor groups varied in their electron‐donating abilities. The study indicated that the looser arrangement of MAP3‐FE and MAP4‐FE in an aggregated state provided more space for molecular movement. Compared to MAP3‐FE, MAP4‐FE with a smaller substituent exhibited greater bond rotational freedom, resulting in a PCE of 72%. Irradiating MAP4‐FE NPs aggregated at tumor sites in mice with a 660 nm laser almost completely eliminated the tumors within 4 days.^[^ ^157^ ^]^ These findings suggested that the “barrier‐free rotation” strategy presented a promising direction for the future development of SCOPAs.

#### 3.3.4 Photoinduced Nonadiabatic Decay

Lu et al. utilized the concept of photoinduced nonadiabatic decay (PIND) to create a new photothermal molecular core called BOINPY. By conducting structural modifications, compounds with high PCE were successfully obtained (Figure 12b).^[^ ^158^ ^]^ The authors initially observed, through theoretical calculations, that substituting isoindolin‐1‐one with indolin‐3‐one to form BOINPY led to significant changes in its properties, such as a noticeable decrease in energy gap and a spectral redshift. Upon laser excitation, BOINPY in its excited state experienced a strong intramolecular charge transfer effect. The energy difference between S_1_min and the conical intersection (CI) was found to be 0.87 eV, facilitating the effective conversion of light energy into thermal energy via the PIND process at the CI. In contrast, BOISPY exhibited a larger energy gap from S_1_min to CI (1.75 eV), resulting in intense fluorescence emission due to strong radiative decay. BOINPY@F127 NPs showed good biocompatibility and achieved a PCE of 44.2%, demonstrating significant antitumor therapeutic effects. The Li’ group synthesized a pair of imine‐based and vinyl‐based molecular motors (Figure 12c). The imine‐based molecular motor, unlike its vinyl‐based counterpart, displayed minimal fluorescence in both liquid and solid states owing to the enhanced intramolecular motion facilitated by double bond torsion, leading to a photothermal effect. Notably, the absorption of the imine‐based molecular motor could be red‐shifted into the NIR region due to the strong twisted intramolecular charge transfer (TICT) effect. This red‐shift further enhanced photothermal conversion under 808 nm laser irradiation, achieving a maximum PCE of around 90.0%, significantly surpassing most reported photothermal agents, including ICG. The strategy avoided the need for long alkyl chains or complex substituents in traditional organic photothermal agent designs, providing a new approach for the development of SCOPAs.^[^ ^159^ ^]^

#### 3.3.5 Twisted Intramolecular Charge Transfer

TICT, a photophysical phenomenon commonly observed in conjugated systems with a D‐A structure, occurs when a molecule absorbs energy and enters an excited state, easily transitioning into the TICT state under polar conditions. Molecules in the TICT state primarily dissipate energy and return to the ground state through nonradiative transitions. Molecular motion is a prerequisite for forming the TICT state. However, for in vivo applications, SCOPAs are typically encapsulated in NPs to restrict intramolecular rotation in the tightly aggregated state, inhibiting the TICT state formation. To promote the TICT transition, Han et al. co‐assembled the photothermal agent BTPPA with an amphiphilic copolymer into NPs. These NPs remained stable under neutral conditions but could disassemble and release BTPPA in an acidic environment. The released BTPPA transitioned from an aggregated to a dispersed state, enhancing rotational capacity and promoting TICT state formation. As a result, the corresponding PCE increased from 43% to 60%. Conformational calculations revealed that these molecules exhibited highly twisted conformations when existing independently, allowing free rotation of triphenylamine and facilitating the transition to the TICT state. In in vivo experiments, after injecting these NPs and irradiating them with a 660 nm laser (0.3 W cm^−2^), which increased the temperature by 15 °C within 3 min, tumors were eliminated completely three days later, demonstrating excellent PTT effects.^[^ ^160^ ^]^

### 3.4 Multimodal Synergistic Treatments Based on PTT

While monomodal PTT has demonstrated significant results, it still faces some thorny issues. One notable limitation is the restricted penetration depth of NIR laser, resulting in effective treatment primarily for superficial skin tumors. Deeper tumors may not be completely eradicated, increasing the risk of recurrence and metastasis. Despite these limitations, the thermal effects of PTT also offer various functional benefits, such as regulating drug release, activating enzymes, stimulating anti‐tumor immune responses, and enhancing chemical reactions in targeted tissues.^[^ ^161^ ^]^ Combining PTT with other treatment modalities like chemotherapy,^[^ ^162^ ^]^ radiotherapy,^[^ ^163^ ^]^ gene therapy,^[^ ^164^ ^]^ sonodynamic therapy,^[^ ^165^ ^]^ and starvation therapy can provide a more effective strategy to enhance anti‐tumor efficacy.^[^ ^166^, ^167^ ^]^

#### 3.4.1 PTT Combined with Chemotherapy

**Figure 13:**
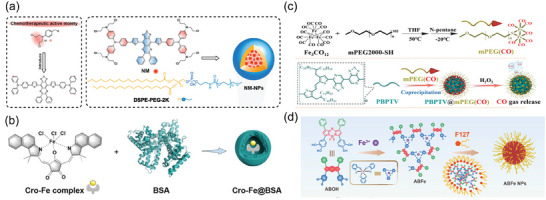
a) Design of single‐molecule NM‐based nano‐platform for NIR‐II imaging and photo‐chemo‐therapy. Reproduced with permission.^[^ ^170^ ^]^ Copyright 2022, Elsevier. b) Fabrication of Cro‐Fe@BSA nanoparticles for ferroptosis and the PTT. Reproduced with permission.^[^ ^173^ ^]^ Copyright 2022, Wiley‐VCH. c) synthesis route of mPEG(CO) and  the nanobomb consist of mPEG(CO) and PBPTV for CO‐assisted PTT. Reproduced with permission.^[^ ^174^ ^]^ Copyright 2022, Wiley‐VCH. d) Preparation for ABFe NPs for photothermal‐enhanced chemodynamic therapy. Reproduced with permission.^[^ ^180^ ^]^ Copyright 2023, American Chemical Society.

Cheng's team successfully developed an NIR‐II SCOPA with a D‐A‐D structure, which features 6, 7‐diphenylthiadiazolequinoline as the electron acceptor and TPA as the electron donor. Moreover, by incorporating the active fragment bis‐(dichloroethyl) amino from nitrogen mustard drugs onto TPA, researchers aimed to leverage the tumor‐killing properties of nitrogen mustard compounds.^[^ ^168^, ^169^ ^]^ The resulting compound, NM, was further encapsulated into NM NPs coated with DSPE‐PEG2000. Experimental findings demonstrated that NM NPs exhibited both NIR‐II fluorescence and a high PCE of 47.38%, while also displaying chemotherapeutic effects. Combining PTT and chemotherapy in NM NPs effectively inhibits tumor growth. This innovative design not only integrates NIR‐II fluorescence, photothermal, and chemotherapy functionalities into SCOPAs but also sets a promising direction for future integrated diagnostic probe development (**Figure** **13**a).^[^ ^170^ ^]^

#### 3.4.2 PTT Combined with Ferroptosis

As a novel mode of cell death, ferroptosis is widely used in cancer treatment. Especially when tumor cells are insensitive to other death pathways such as apoptosis and necrosis, ferroptosis can still be effectively induced to kill tumor cells. Therefore, ferroptosis has shown great advantages in cancer treatment.^[^ ^171^, ^172^ ^]^ Starting from the inherent mechanisms of both PTT and ferroptosis therapy, Zhou et al. constructed a nanotherapeutic platform (Cro‐Fe@BSA) based on croconic acid.^[^ ^173^ ^]^ The croconic acid molecules encapsulated within bovine serum albumin acted as both a ferric ion chelating agent and a photothermal converter, capable of inducing the photothermal effect in the tumor TME. Simultaneously, the ligand iron was converted into highly reactive ferrous iron by the reduction of GSH, initiating the Fenton reaction. This dual mechanism not only enhanced the accumulation of intracellular reactive iron pool (ferrous iron) but also reduced the level of GPX4, ultimately triggering ferroptosis in cells. The photothermal effect generated by nanomaterials not only functions for PTT but also accelerates the Fenton reaction, thereby improving the efficacy of ferroptosis in killing tumor cells. Additionally, the intracellular accumulation of highly reactive •OH and lipid peroxidation can inhibit the expression of HSP70, a heat shock protein that confers resistance to photothermal therapy, thus eliminating its protective effects on tumor cells, exhibiting superior tumor suppression in breast cancer tumor model (Figure 13b).

#### 3.4.3 PTT Combined with Gas Therapy

Due to the upregulation of heat shock proteins (HSPs), the effect of low‐temperature tumor ablation is insufficient. Gong et al. developed a nanobomb (PBPTV@mPEG(CO)) that released CO to suppress HSPs, disrupting tumor thermoresistance and promoting cell death at 43 °C without compromising normal cells (Figure 13c).^[^ ^174^ ^]^ Hu's team synthesized the first NIR J‐aggregate (BDP‐NO) for NO release by nitrosation of the amino‐containing aza‐BODIPY precursor (BDP‐NH_2_). Experimental findings showed that the combined NO and PTT approach enhanced antibacterial efficacy and mitigated PTT‐related inflammation in the treatment of MRSA infections in vivo.^[^ ^175^ ^]^ Liu et al. designed a versatile platform capable of synergistic NO and PTT effects using an aza‐BODIPY skeleton (S‐NO) functionalized with aryl N‐nitrosamine (NO donor). S‐NO NPs effectively suppressed tumor growth and enhanced mouse survival rates through synergistic NO and PTT effects.^[^ ^176^ ^]^ Yang's team developed a SCOPA platform (IR‐FEP‐RGD‐S‐S‐S‐Fc) that released H_2_S to inhibit mitochondrial cytochrome c oxidase, leading to mitochondrial dysfunction, decreased ATP synthesis, and enhanced HPTT efficiency.^[^ ^177^ ^]^

#### 3.4.4 PTT Combined with CDT

CDT converts intracellular H_2_O_2_ into toxic ROS through Fenton or Fenton‐like reactions.^[^ ^178^ ^]^ It is known for its specific response to the TME, high selectivity, and low risk of inducing tolerance. However, its effectiveness is limited by the concentration of H_2_O_2_ and the slow rate of the Fenton reaction. Research has shown that increasing the temperature in the tumor area can accelerate the catalytic Fenton reaction, thus improving the therapeutic outcomes of CDT.^[^ ^179^ ^]^ Yin et al. developed NIR‐II‐excited SCOPAs called ABFe NPs for the combined treatment of PTT and CDT. They utilized F127 to encapsulate a 3D metal‐polyphenol network created by coordinating metal‐multidentate ligands modified with iron (III) and catechol‐modified aza‐BODIPY to form ABFe NPs. The introduction of iron ions induced a ligand‐to‐metal electron transfer effect, extending the absorption wavelength of ABFe NPs into the NIR‐II region (300–1300 nm), enhancing photothermal conversion under NIR‐II laser irradiation. In vivo experiments showed that under NIR‐II laser irradiation, ABFe NPs displayed excellent photoacoustic imaging capabilities and synergistic PTT‐enhanced CDT therapeutic effects, leading to significant inhibition of tumor growth (Figure 13d). This study offered valuable insights into the development of SCOPAs based on metal‐organic complexes.^[^ ^180^ ^]^

#### 3.4.5 PTT Combined with PDT Induced Immunotherapy

**Figure 14:**
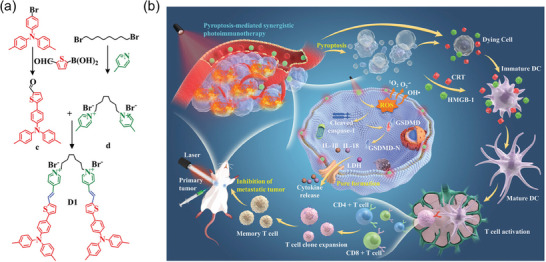
a) Synthesis of photosensitive dimer D1. b) Schematic illustration of pyroptosis‐mediated photothermal/photodynamic immunotherapy enabled by D1 and its inhibition effect of metastatic tumor demonstrated by intratumoral injection of primary tumor. Reproduced with permission.^[^ ^183^ ^]^ Copyright 2023, Wiley‐VCH.

PTT and PDT can induce ICD of cancer cells, releasing CRT, HMGB1, and ATP extracellularly, thereby recruiting immune cells.^[^ ^181^, ^182^ ^]^ Based on this, Li's team presented a tumor cell membrane‐targeted AIE photosensitizer dimer (D1) capable of achieving efficient ICD through the combined effects of PDT and PTT (**Figure** **14**a). This dimer could generated type‐I ROS effectively in hypoxic tumor tissue, inducing pyroptosis by directly damaging the cell membrane, while the photothermal effect further intensified the pyroptosis process. The synergistic phototherapy‐induced pyroptosis facilitated the release of cellular contents and inflammatory cytokines, triggering an anti‐tumor immune response. It stimulated the production of tumor‐specific antigens and dendritic cell maturation, leading to activated T cell proliferation, establishing systemic anti‐tumor immunity and creating significant immune memory (Figure 14b). This study provided a new idea for photoimmunotherapy of tumors.^[^ ^183^ ^]^ The applications of PTT induced by SCOPAs have been summarized in **Table** **2**.

**Table 2:** The applications of PTT induced by SCOPAs.

Name	Abs [nm]^a)^	Laser [nm]	Power [W cm^−2^]	PCE [%]	Strategy	Ref.
DCP‐TPA	610	660	0.45	52	3.1.1	[128]
DCP‐PTPA	610	660	0.45	59	3.1.1	[128]
2TPE‐NDTA	670	808	0.8	43	3.1.2	[129]
2TPE‐2NDTA	760	808	0.8	54.9	3.1.2	[129]
M2‐NPs	598	660	0.75	47.2	3.1.3	[131]
TDADT	838	808	0.5	60.4	3.1.3	[132]
Cy7‐TCF‐EMBI	852	808	1	63.3	3.1.4	[133]
OIS	817	808	0.8	/	3.2.1	[140]
PIS	1026	1064	1	/	3.2.1	[140]
N1@2P	900	1064	1	55.6	3.2.2	[141]
FTQ	1140	1064	1	49	3.2.2	[142]
BETA	873	1064	1	47.6	3.2.3	[143]
TTQ‐2TC‐4T	817	1064	1	44.43	3.2.4	[145]
OSP12	857	1064	1	45.25	3.2.4	[146]
DPQ	1015	1064	1	40.92	3.2.4	[147]
PER‐TCNQ	1040	1064	1	42	3.2.5	[150]
PSQ	745	808	0.3	81.2	3.3.1	[151]
BisBDP2	1273	1208	0.8	63	3.3.2	[155]
tfm‐BDP	810	808	0.3	88.3	3.3.3	[156]
MAP4‐FE	590	660	1	72	3.3.3	[157]
BOINPY	650	650	0.5	44.2	3.3.4	[158]
C6TI	750	808	1	89.3	3.3.4	[159]
BTPPA	620	660	0.3	60	3.3.5	[160]
NM	850	808	1	47.38	3.4.1	[170]
Cro‐Fe@BSA	810	785	1	17.6	3.4.2	[173]
PBPTV@mPEG(CO)	810	808	/	38.1	3.4.3.1	[174]
BDP‐NO	820	808	0.375	48.5	3.4.3.2	[175]
S‐NO	702	808	0.5	/	3.4.3.2	[176]
IR‐FEP‐RGD‐S‐S‐S‐Fc	770	808	1	41.7	3.4.3.3	[177]
ABFe	800	1064	1	55.0	3.4.4	[180]
D1	497	520	0.3	/	3.4.5	[183]

^a)^ The absorption wavelength of SCOPAs.

## 4 SCOPAs Simultaneously Achieving PDT and PTT

PDT and PTT have seen significant development in the past decade, emerging as promising therapeutic approaches. However, they both have limitations. As a result, efforts have shifted toward combined PDT and PTT as a more rational treatment strategy. PDT can reduce HSP overexpression during PTT through ROS, enhancing PTT effectiveness. Conversely, PTT can use thermal energy to increase molecular movement in tumor cells, boosting oxygen levels around them and reducing the oxygen dependency of PDT, thereby enhancing its effects. Currently, most combined PDT and PTT treatment strategies are complex, which hinders further clinical applications.^[^ ^184^ ^]^ Therefore, there is a growing interest in developing SCOPAs that can achieve synergistic therapeutic effects of both PDT and PTT in the biomedical field.

### 4.1 Single Electron Acceptor Adjustment

**Figure 15:**
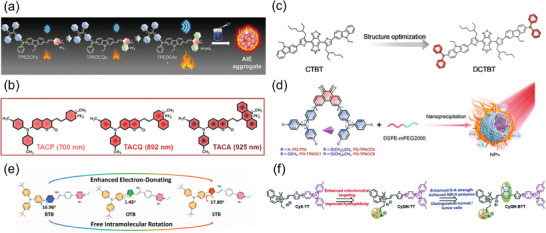
Schematic diagram of SCOPAs structures. a) TPEDCPy, TPEDCQu and TPEDCAc. Reproduced with permission.^[^ ^185^ ^]^ Copyright 2022, Wiley‐VCH. b) TACP, TACQ, and TACA. Reproduced with permission.^[^ ^186^ ^]^ Copyright 2021, American Chemical Society. c) CTBT and DCTBT. Reproduced with permission.^[^ ^187^ ^]^ Copyright 2022, Elsevier. d) PQ‐TPA, PQ‐TPAOC4, PQ‐TPAOC1 and PQ‐TPAOC8. Reproduced with permission.^[^ ^188^ ^]^ Copyright 2021, American Chemical Society. e) BTB, OTB, and STB. Reproduced with permission.^[^ ^191^ ^]^ Copyright 2023, American Chemical Society. f) TI, TSI and TSSI. Reproduced with permission.^[^ ^192^ ^]^ Copyright 2020, Wiley‐VCH.

The modulation of energy dissipation in excited SCOPAs to achieve a balance between PDT and PTT remains a challenging task. Lin and Zhao's team implemented a molecular engineering strategy by introducing strong electron acceptors such as positively charged pyridinium, quinolinium, and acridinium salts into the molecular system. This led to the creation of a series of SCOPAs with similar structures (**Figure** **15**a,b).^[^ ^185^, ^186^ ^]^ In Lin's research, the TPEDCAc molecule, which exhibited AIE characteristics, demonstrated good NIR‐II luminescence properties, high ROS generation ability, and an impressive PCE of up to 44.8%. Both experimental and theoretical calculations indicated that the positively charged acridinium salt could induce a significant ICT effect, while the bulky group size and torsion angle enhanced intramolecular motion. As a result, the TPEDCAc molecule effectively balanced radiative and nonradiative transition processes, showcasing excellent NIR‐II luminescence properties, as well as superior photodynamic, photoacoustic, and photothermal conversion.

### 4.2 Single Electron Donor Engineering

The Ji and Zhao groups utilized a molecular engineering approach to enhance both PDT and PTT effects by manipulating electron donors (Figure 15c‐d).^[^ ^187^, ^188^ ^]^ They developed an AIE photosensitizer, DCTBT, with NIR‐II fluorescence emission by incorporating diphenylamine groups at both ends of the D‐A type conjugated small molecule skeleton. The diphenylamine groups not only increased the AIE characteristics of DCTBT by providing molecular rotors but also enhanced intramolecular charge transfer interactions. This led to a red shift in absorption and emission wavelengths of DCTBT, along with a smaller ΔE_S‐T_, ultimately improving ROS generation efficiency and PCE. Guo and Tang et al. also synthesized various SCOPAs with combined photodynamic and photothermal properties using a donor‐acceptor regulation strategy for synergistic diagnosis and treatment of tumors.^[^ ^189^, ^190^ ^]^

### 4.3 Single π Bridge Regulation Guidance

Our research group developed a series of SCOPAs utilizing benzene rings, furans, and thiophenes as π bridges through a strategy known as π bridge engineering. The goal was to achieve a synergistic type‐I PDT and PTT for targeting tumor mitochondria. By controlling the electron‐donating ability of the π‐bridge and enabling intramolecular free motion, the photosensitizer STB, with thiophene as the π bridge, displayed exceptional O_2_^•−^ and •OH generation capabilities along with significant photothermal conversion capabilities. Theoretical calculations indicated that STB exhibited strong D‐A strength, which aided in reducing ΔE_S‐T_, enhancing ISC, and promoting PDT. Moreover, the higher molar extinction coefficient and flexible dihedral angle rotation facilitated intramolecular motion, resulting in an impressive PCE of up to 51.9% for STB. It is noteworthy that the positively charged STB could effectively target tumor cell mitochondria through electrostatic interactions, leading to enhanced accumulation in the tumor area and improved therapeutic outcomes. Both in vitro and in vivo experiments confirmed that STB NPs significantly suppressed cancer cell proliferation and tumor growth. This study offered valuable insights into designing and developing tumor‐specific targeted multifunctional SCOPAs for precise cancer phototherapy (Figure 15e).^[^ ^191^ ^]^ Peng’ team synthesized a range of D‐π‐A type SCOPAs (CyE‐TT, CyQN‐TT, and CyQN‐BTT) utilizing quaternized 1, 1, 2‐trimethyl‐1H‐benz[e]indoles as acceptors (Figure 15f). They extended the emission wavelength by introducing a π‐bridge and using triphenylamine as a donor to create a twisted molecular conformation. The researchers observed a direct correlation between the ^1^O_2_ generation ability and PCE with the π‐bridge in these molecules. Upon incorporating the 2, 1, 3‐benzothiadiazole group as a π‐bridge into CyQN‐BTT, the ^1^O_2_ yield increased to 27.1%, and the PCE rose to 37.8%.^[^ ^192^ ^]^ Based on the π‐bridge engineering strategy, the Tang research group has also successfully developed multiple series of multifunctional SCOPAs by varying the type and number of π‐bridges.^[^ ^193^, ^194^ ^]^

### 4.4 Regulating Energy Dissipation Pathways

Regulating energy dissipation pathways through rational structural design is an effective strategy for preparing SCOPAs with both PDT and PTT capabilities.^[^ ^195^, ^196^, ^197^, ^198^, ^199^ ^]^ Li et al. synthesized two molecules, TPE‐TBT and TPA‐TBT, with strong AIE properties by modifying electron‐donating and electron‐withdrawing groups. The electron‐withdrawing group TBT, known for its strong electron‐withdrawing ability and chemical stability, was connected to alkyl‐substituted thiophene to prevent intermolecular π‐π stacking. Methoxy TPE and TPA groups were used as electron‐donating units, and alkylated thiophene as the π‐bridge, resulting in a twisted backbone structure for improved AIE properties and solubility. While TPE‐TBT showed high fluorescence quantum yield, its nonradiative transition energy limited its potential for PDT and PTT applications. On the other hand, TPA‐TBT displayed a significant red shift in absorption and emission spectra due to its charge transfer effect and twisted conformation. Nano‐aggregates of TPA‐TBT exhibited bright fluorescence, high PCE, and ROS generation. These properties enabled a balance between radiative and nonradiative energy dissipation, facilitating synergistic diagnosis and treatment through PDT and PTT.^[^ ^200^ ^]^

### 4.5 Conformation Design

**Figure 16:**
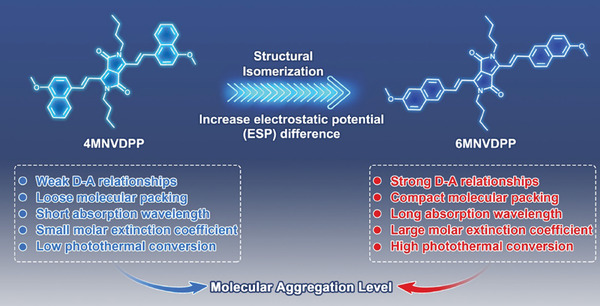
Design strategy for preparing NIR‐II phototherapeutic agents by controlling the molecular packing of 4MNVDPP and 6MNVDPP. Reproduced with permission.^[^ ^201^ ^]^ Copyright 2022, Wiley‐VCH.

Among the reported NIR‐II SCOPAs, common molecular design strategies are focused on extending the π‐conjugation length or enhancing D‐A interactions, despite their typically poor water solubility. Matrix encapsulation technology is often used to form aggregated NPs, improving biocompatibility and water solubility. However, a literature gap exists regarding the design strategy for constructing NIR‐II SCOPAs by effectively regulating molecular packing during the encapsulation process. Wang et al. conducted research on the relationship between molecular structure and packing, proposing a novel structure isomerization‐induced 3D spatial D‐A interlocking network strategy.^[^ ^201^ ^]^ They synthesized two D‐A‐D type structural isomers, 4MNVDPP and 6MNVDPP, and achieved different packing modes by utilizing their distinct surface electrostatic potentials (**Figure** **16**). 6MNVDPP exhibited a tight 3D spatial D‐A interlocking network packing in the crystal, while 4MNVDDP formed a 2D D–D type J‐aggregated packing. Under 808 nm laser irradiation, 6MNVDPP NPs showed a PCE of 89% and excellent ROS generation capability, enabling NIR‐II‐FLI‐guided PDT‐PTT synergistic therapy. Lee et al. expanded the intramolecular donor via molecular design to enhance D‐A conformational distortion and ICT effects, preparing the molecule BT6. This strategy effectively tackled the self‐quenching problem often observed in J‐aggregated fluorophores, leading to an increased Stokes shift and anti‐quenching properties. The molecule was then incorporated into a water‐soluble nanomedicine using nano‐formulation technology, capable of producing a high amount of ROS and heat when exposed to 808 nm laser irradiation. This research introduced a novel molecular design concept for enhancing NIR‐II fluorescence emission in J‐aggregated molecules by adjusting the donor structure, providing innovative approaches for bioimaging and cancer phototherapy.^[^ ^202^ ^]^ The applications of PDT and PTT achieved by SCOPAs have been summarized in **Table** **3**.

**Table 3:** The applications of PDT and PTT achieved by SCOPAs.

Name	Abs [nm]^a)^	Laser [nm]	Power [W cm^−2^]	ROS type	PCE [%]	Strategy	Ref.
TPEDCAc	580	660	0.3	^1^O_2_, •OH, O_2_^•−^	44.8	4.1	[185]
TACQ	510	635	0.6	^1^O_2_	55	4.1	[186]
DCTBT	720	808	0.8	•OH, O_2_^•−^	59.6	4.2	[187]
PQ‐TPAOC1	540	660	0.8	•OH	37.1	4.2	[188]
CySe‐NEt2	776	750	0.5	^1^O_2_	42.2	4.2	[189]
MeTIC	632	660	0.3	^1^O_2_	34.8	4.2	[190]
STB	480	532	0.2	•OH, O_2_^•−^	51.9	4.3	[191]
CyQN‐BTT	677	671	0.4	^1^O_2_	37.8	4.3	[192]
TSSI	640	660	0.3	•OH	46	4.3	[194]
Py‐PS	620	808	1	•OH, O_2_^•−^	86.8	4.4	[195]
Y16‐Pr	780	808	1	•OH ^1^O_2_	82.4	4.4	[196]
BHcy	770	808	1.5	^1^O_2_	55.1	4.4	[197]
TTT‐4	568	660	0.3	•OH ^1^O_2_	39.9	4.4	[198]
BITT	575	660	0.3	^1^O_2_, OH, O_2_^•−^	35.76	4.4	[199]
TPA‐TBT	620	660	1	/	44	4.4	[200]
6MNVDPP	764	808	0.5	O_2_^•−^	89	4.5	[201]
BT6	652	808	1	/	36	4.5	[202]

^a)^ The absorption wavelength of SCOPAs.

## 5 Challenges and Prospects of SCOPAs

To make a greater contribution in the fields of PDT and PTT, SCOPAs still need to address the following significant issues.

1) Limited penetration: The absorption and scattering effects of human tissues on laser light gradually increase as the laser propagates through the tissue, ultimately leading to a rapid attenuation of laser energy and a limited penetration depth. Thus, the treatment depth of PDT and PTT is also restricted. To overcome this challenge, molecular design and skin clearing strategies were proposed. In terms of molecular design, developing materials with absorption in the NIR‐II region or beyond, utilizing upconversion materials, and exploring multiphoton photodynamic are main methods.^[^ ^203^, ^204^, ^205^ ^]^ In addition, skin‐clearing techniques can be also as a complementary approach, which involves the application of chemical agents or physical methods to temporarily reduce the scattering and absorption properties of the skin, thereby enhancing light penetration.^[^ ^206^ ^]^ Especially for PDT, employing self‐iluminescence activated SCOPAs is also a top option. Self‐luminescence can avoid the limitation of light penetration depth, thereby improving the effectiveness of PDT.

2) Intratumoral enrichment: Ideal SCOPAs should specifically accumulate in tumor tissues to reduce damage to normal tissues. However, the tumor targeting of many SCOPAs still needs improvement. In addition to modifying specific tumor‐targeting molecules such as antibodies, peptides, or small molecules on the surface of NPs to achieve active targeting, there are several other approaches that can help enhance the enrichment of SCOPAs within tumor cells. For instance, by chemically bonding or physically adsorbing specific antibodies to SCOPAs, the SCOPAs can specifically recognize and bind to antigens on the surface of tumor cells, thereby improving their targeting ability. Designing SCOPAs sensitive to the characteristics of the TME, such as hypoxia and low pH, can also enhance targeting to tumor tissues. Furthermore, enabling the SCOPAs to self‐assemble in situ to form aggregates within tumor cells can improve the enrichment degree of nanomedicines. For example, by modifying amino acids on the structure of the SCOPAs, the amino acid molecules can cross‐link with each other under the action of biological enzymes to form stable and ordered macromolecular aggregates in situ, reducing efflux and achieving high accumulation within the tumor.

3) Higher biocompatibility: It is necessary to further enhance the Stability and solubility of the SCOPAs. Chemical modifications on the surface of NPs, such as introducing hydrophilic groups like sulfonic acid and carboxylic acid groups, can increase the compatibility of NPs with water molecules, thereby improving their stability in water. Encapsulating the SCOPAs within hydrophilic materials such as amphiphilic polymers or liposomes can enhance its dispersibility and solubility in water. The last but not least, preliminary studies indicate that SCOPAs typically demonstrate good biocompatibility. However, for clinical translation, it is essential to gather systematic and comprehensive in vivo study data, covering aspects such as pharmacokinetics, biodistribution, metabolism, excretion, and a thorough evaluation of long‐term safety. This necessitates extensive collaboration among multiple stakeholders.

Even though SCOPAs face some significant and essential issues that must be addressed in their further development, they still hold great promise for future clinical applications. Compared with an “all in one” strategy, which involves assembling multiple molecules with different functions into a single nanoparticle to achieve enhanced therapeutic effects. This approach faces challenges such as complex synthesis, high cost, poor reproducibility, mutual interference between components, and unclear pharmacokinetics, making clinical translation a rigorous process. SCOPAs with “one for all” capabilities eliminates the need to consider interactions between multiple components.^[^ ^207^ ^]^ The preparation process is simpler, more efficient, and cost‐effective, with strong reproducibility. Additionally, compared to inorganic nanomaterials, SCOPAs offer more diverse structures, flexible designs, good solubility, and strong processability. Importantly, they exhibit better biocompatibility, lower potential toxicity, and are easier for the body to metabolize and excrete, demonstrating superior biosafety. Therefore, they have greater potential for clinical translation. To achieve the multifunctional synergistic therapies for SCOPAs, the key lies in regulating their structures. For PDT: 1) Considering stronger D‐A properties in the molecular skeleton to ensure a small ΔE_S‐T_; 2) Introducing the heavy atoms to increase the SOC; 3) Achieving type‐I PDT by reducing the T1 energy of the SCOPAs below the oxygen sensitization threshold of 0.98 eV; 4) Developing SCOPAs with an A‐D‐A structure for stronger photodynamic performance; For PTT: 1) Importing free rotating rotors to strengthen molecular motion; 2) Extending conjugated structures for a larger molar extinction coefficient; 3) Inhibiting radiation transitions pathway to promote photothermal conversion.

## 6 Conclusion

Given the long‐term potential toxicity associated with inorganic nanomaterials, the development of organic phototherapeutic agents with superior biocompatibility has become imperative. Traditional organic phototherapeutic agents, however, grapple with challenges such as inadequate ROS generation and suboptimal photothermal conversion properties. Therefore, an “all in one” strategy has emerged as a promising approach, albeit with hurdles including complex synthesis processes, poor reproducibility, high costs, and unclear pharmacokinetics that impede clinical application. Considering that, there is an urgent need to develop SCOPAs with excellent photodynamic or photothermal performance.

Structure determines performance. In this review, several subtle strategies that can improve photosensitization or photothermal conversion are systematically summarized. In the PDT section, we mainly introduced: 1) Strategies to improve type‐II PDT; 2) Methods to promote type‐I PDT; 3) Design rules for both type‐I and type‐II PDT. In the PTT section, we primarily discussed: 1) Approaches to strengthen NIR‐I photothermal conversion; 2) Principles for constructing NIR‐II SCOPAs; 3) Ideas for designing SCOPAs with ultrahigh PCE. Finally, we showcased the combination of PDT and PTT with other treatments, as well as how to achieve a balanced PDT and PTT performance in SCOPAs.

Overall, SCOPAs have demonstrated remarkable therapeutic efficacy in various disease models, both in vitro and in vivo, offering an additional avenue for disease treatment. Based on their dazzling advantages and promising research results, we can reasonably predict the great possibility of clinical transformation of SCOPAs in the future. We hope that this review can stimulate interest in collaborative research in related interdisciplinary fields, working together in this passionate and promising research area to promote the transition of therapeutics and diagnostics based on SCOPAs toward broad clinical applications.

## Conflict of Interest

The authors declare no conflict of interest.
